# Characterizing the Spatial Determinants and Prevention of Malaria in Kenya

**DOI:** 10.3390/ijerph16245078

**Published:** 2019-12-12

**Authors:** Sucharita Gopal, Yaxiong Ma, Chen Xin, Joshua Pitts, Lawrence Were

**Affiliations:** 1 Department of Earth & Environment, Boston University, Boston, MA 02215, USA; suchi@bu.edu (S.G.); yxma@bu.edu (Y.M.); xinch963@bu.edu (C.X.); 2 Center for Global Development Policy, Boston University, Boston, MA 02215, USA; joshua.d.pitts@gmail.com; 3 College of Health & Rehabilitation Sciences: Sargent College, Boston University, Boston, MA 02215, USA

**Keywords:** hot spot analysis, spatial autocorrelation, geographically weighted regression, malaria, spatial non-stationarity, principal component analysis, Kenya

## Abstract

The United Nations’ Sustainable Development Goal 3 is to ensure health and well-being for all at all ages with a specific target to end malaria by 2030. Aligned with this goal, the primary objective of this study is to determine the effectiveness of utilizing local spatial variations to uncover the statistical relationships between malaria incidence rate and environmental and behavioral factors across the counties of Kenya. Two data sources are used—Kenya Demographic and Health Surveys of 2000, 2005, 2010, and 2015, and the national Malaria Indicator Survey of 2015. The spatial analysis shows clustering of counties with high malaria incidence rate, or hot spots, in the Lake Victoria region and the east coastal area around Mombasa; there are significant clusters of counties with low incidence rate, or cold spot areas in Nairobi. We apply an analysis technique, geographically weighted regression, that helps to better model how environmental and social determinants are related to malaria incidence rate while accounting for the confounding effects of spatial non-stationarity. Some general patterns persist over the four years of observation. We establish that variables including rainfall, proximity to water, vegetation, and population density, show differential impacts on the incidence of malaria in Kenya. The El-Nino–southern oscillation (ENSO) event in 2015 was significant in driving up malaria in the southern region of Lake Victoria compared with prior time-periods. The applied spatial multivariate clustering analysis indicates the significance of social and behavioral survey responses. This study can help build a better spatially explicit predictive model for malaria in Kenya capturing the role and spatial distribution of environmental, social, behavioral, and other characteristics of the households.

## 1. Introduction

Malaria is one of the leading causes of morbidity and mortality in the world, with an estimated 219 million incidences and 435,000 deaths worldwide in 2017 [1]. About 92% of malaria incidence in 2017 was in sub-Saharan Africa [1]. Children aged under five years are the most vulnerable group accounting for 61% of all malaria deaths worldwide, with the African region accounting for 93% of all malaria deaths in 2017 [1]. Malaria is caused by the parasite *Plasmodium* that is transmitted to human hosts through a vector, the infected female *Anopheles* mosquitoes [2]. The two species of *Plasmodium*—*P. falciparum* (Africa and SE Asia) and *P. vivax* (Americas) pose the greatest threat, while a diverse group of Anopheles (30 to 40 species) serves as vectors for this disease vector biology [2]. Malaria symptoms can vary from headache, fatigue, body aches, nausea, and vomiting to severe complications such as “cerebral malaria/coma, seizures, severe anemia, respiratory distress, kidney and liver failure, cardiovascular collapse, and shock”, in vulnerable groups consisting of children and pregnant women [3].

The focus of the United Nations’ Sustainable Development Goal 3 is to ensure health and well-being for all at all ages; a more specific goal is to reduce the disease burden and eliminate malaria by 2030 [4]. Malaria is a significant social and economic burden since it leads to deaths, as well as limits economic development as substantial fractions of funds are spent on malaria control and treatment in countries impacted by the disease [5,6]. It is therefore critical to analyze malaria on a population level and determine the environmental, social, behavioral factors that influence malaria epidemiology and transmission. In this paper, we focus on malaria in Kenya, analyzing population-level, spatial determinates of the disease using data from the time period 2000–2015.

About 70 percent of Kenya’s population lives in malaria risk areas, including a vulnerable population of children and pregnant women [7]. Kenya is also one of the 15 high-burden countries in sub-Saharan Africa that are part of the President’s Malaria Initiative launched in 2005 to reduce malaria-related mortality by 50% [8]. According to the Kenya National Bureau of Statistics (KNBS) in 2016, malaria was the second leading cause of mortality in Kenya, accounting for 8% of the total mortality incidence. Different studies in Kenya have looked at the prevalence, determinants, and outcomes of malaria among children [9,10,11,12,13,14,15], malaria endemicity and vector abundance [16,17], the costs/cost-effectiveness of malaria control interventions [18,19], the socioeconomics and epidemiology of malaria in western Kenya [20,21,22], and the effectiveness of different interventions for malaria control and prevention [23,24,25,26,27,28]. Additional studies have analyzed the relationship between climate and malaria in the western and coastal regions of the country [29,30]. The Kenya Health Policy 2014–2030 “Towards Attaining the Highest Standard of Health” [31] notes that interventions such as the increased use of insecticide treated nets (ITNs), intermittent prophylaxis treatment (IPT), and indoor residual spraying (IRS) are leading to a reduction in malaria infections. A further reduction of malaria risk may be possible by analyzing its spatial distribution using nationally representative data and identifying temporal changes in areas of a high risk for malaria. Spatial analysis of the adoption of interventions can also assist in management and targeted deployment of limited resources to areas of need for greatest impact [32,33].

Specific to spatial temporal analysis of malaria, several studies have been undertaken in Africa more broadly and in Kenya including in malaria endemic zones in the western and coastal regions of the country. A recent study [34] analyzed the spatial panorama of malaria prevalence in Africa under climate change and different intervention scenarios. Additional studies have used spatial tools to analyze the prevalence of *Plasmodium falciparum* and malaria vectors across different regions of Kenya [35,36,37,38]. There is growing use of spatial temporal analysis to study malaria risk, transmission, and mortality [39,40,41]. These spatial studies have either used facility or region-specific data but not nationally representative data as we do in our analysis.

Kenya has a diverse ecology consisting of savannah, tropical, equatorial, and volcanic land cover [42]. The most distinct landform is the east African Rift Valley that extends from Lake Turkana to Lake Victoria and further southeast to the Indian Ocean [43]. These highlands are forested compared with the drier and arid regions of the northeast [42]. Both landforms and climate influence the spatial distribution and transmission of malaria [44,45,46]. Moreover, major El Nino–southern oscillation (ENSO) events were recorded in 2015 that are usually associated with wetter and warmer conditions during the short rainy season of October–December [47] and are associated with adverse health effects including malaria [48].

**Figure 1:**
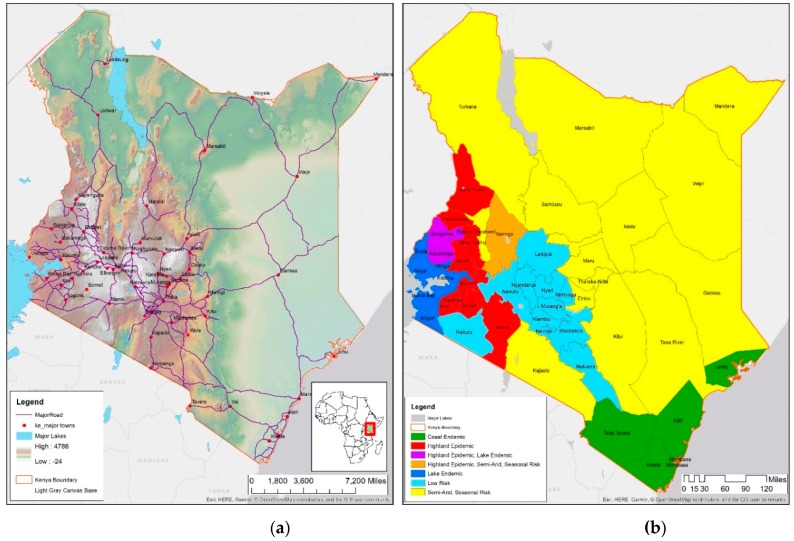
Kenya (**a**) map showing elevation, cities, and road network; (**b**) five malaria zones in Kenya showing counties in each zone.

Figure 1a shows the elevation of Kenya and the major road network. The densest road network is in the south central area around the capital city of Nairobi followed by dense networks towards Lake Victoria in the west. Lowest networks correspond to lower population in the sparsely populated region of the north and the northeast. Figure 1b shows the malaria zones of Kenya [49]. Coast endemic along the east coast that includes (i) counties of Mombasa, and Taita Taveta; (ii) highland epidemic located south central including, Baringo, Trans-Nzoia, Uasin Gishu, and West Pokot; (iii) lake endemic including Kisumu, Busia, Homa Bay, and Kakamega; (iv) low risk including Nairobi, Nakuru, Nyandarua, Nyeri, and Turkana; (v) semi-arid, seasonal risk encompasses most of the northern counties including Garissa, Mandera, Marsabit, and Wajir. Bungoma and Kakamega, both shown in purple on Figure 1b, are in lake endemic as well, low or seasonal risk zones.

In this study, we present an analysis carried out to investigate the spatial distribution of malaria risk factors in Kenya using estimated incidence data, spatial covariates, and survey data provided by demographic and health surveys. To fully address the spatial heterogeneity at a local scale, we performed multiple types of spatial analysis. Our analysis can help to target specific measures to aid vulnerable populations, as well as build a better spatially explicit tool for malaria community preparedness based on risk factors.

## 2. Data and Methods

### 2.1. Data Sources

For this project, two relevant data sets are utilized: (1) the Kenyan Demographic and Health Survey of 2000, 2005, 2010, and 2015, and (2) the third national Malaria Indicator Survey of 2015 [7] comprised of many attributes elaborated upon below.

#### 2.1.1. Kenyan Demographic and Health Surveys (DHS)

A major source of data on health and development for many emerging economies of the world is the United States Agency for International Development (USAID) funded DHS program which provides high-quality and detailed data on individual health outcomes—particularly outcomes related to maternal and child health [50]. DHS are national surveys carried out in a standardized way at a specific time and provide malaria incidence data from households in clusters adjusted for population (rural–urban) and environmental factors and can also be adjusted for climatic factors. The primary sampling unit in the DHS is villages or population “clusters.” Each cluster contains several households within an administrative unit who participated in the survey. Since most of the data included in DHS may contain personally identifiable information and potentially sensitive, the DHS ensures confidentiality of the respondents by geo-scrambling or displacing the spatial coordinates of the cluster by a set distance in urban (up to 2 km) and rural (5–10 km) from the original geo-location [51]. We abstracted data from Kenyan DHS; there were a total of 1612 DHS clusters with spatial coordinates in 2015.

The DHS program has created country specific geospatial covariate datasets (in both raster and vector Geographic Information System (GIS) formats) to facilitate spatial analysis that links survey cluster locations to ancillary data—known as covariates [52]—that contain data on population, climate, environmental, and other factors. Population is derived by the WorldPop project (University of Southampton) in the form of high resolution and easily accessible data on human population [53]. Vegetation data were extracted from MODIS terra satellite; rainfall estimates (RFE) and altitude were retrieved from the Famine Early Warning Systems Network (FEWS) and the Shuttle Radar Topographic Mission (SRTM) data archives [54]. Proximity to water is defined as the geodesic distance of each DHS cluster to either a lake or the coastline that are derived from global datasets based on shoreline and lake datasets [55].

This DHS-covariates database also includes malaria incidence derived from a dataset called “*Plasmodium falciparum* Incidence Rate” which provides the average number of people per year who show clinical symptoms of *Plasmodium falciparum* malaria within the 2 km (urban) or 10 km (rural) buffer surrounding the DHS survey cluster location [52].

In this analysis, we mapped and extracted DHS geospatial covariates of Kenya [56] relating malaria incidence (cases per 1000 people per year) with rainfall (mm), proximity to water (km), vegetation (0~1), elevation (km), and population distribution (population per hectare) data for DHS clusters. We linked the DHS geospatial covariates [50] of survey cluster locations in Kenya to environmental, demographic, and climate data covariates allowing for more advanced spatial analysis of malaria.

**Figure 2:**
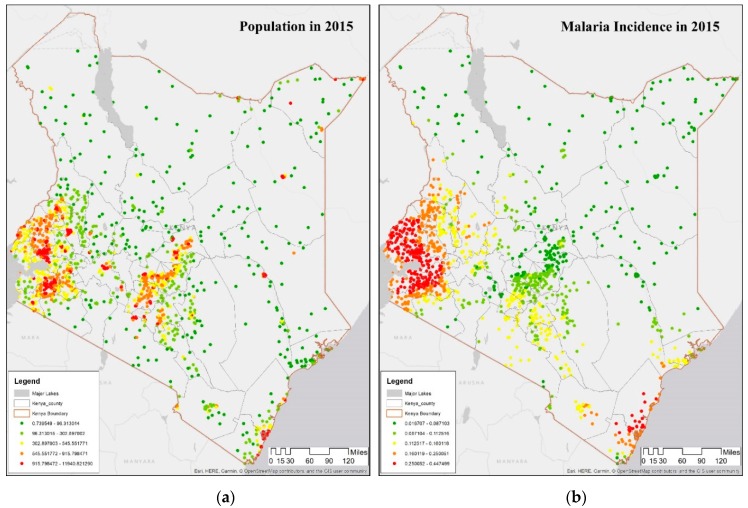
Demographic and Health Surveys (DHS) datasets. (**a**) Map showing population distribution of DHS clusters in counties of Kenya in 2015. (**b**) Map showing average malaria incidence rate per 1000 in DHS clusters in Kenya in 2015. The gray outlines on each map show boundaries of counties in Kenya. On both maps, the transition from red to yellow to green color denotes the change in malaria incidence rate per 1000 from high to low ends of the distribution.

Figure 2a shows the DHS dataset of the 1612 clusters of population, ranging from low (green) to high (red) ends of the distribution, with highest population in the largest cities such as Nairobi, Kisumu, and Mombasa. Figure 2b shows the malaria incidence rate per 1000 data from the covariate database [56], representing the average incidence, i.e., number of individuals per 1000 population per year that showed symptoms. Figure 2b indicates highest values in the malaria incidence are in the southwest around Lake Victoria and the southeastern coast (near Mombasa), both listed as endemic malaria regions in the Kenya Malaria Indicator Survey. The data are included in the spatial models along with four other variables, elevation, rainfall, vegetation, and proximity to water. This DHS dataset of malaria incidence rate per 1000 and co-variates (2000–2015) is used in the hotspot and geographically weighted regression analysis (see Section 3.1 and Section 3.2).

#### 2.1.2. Kenya Malaria Indicator Survey

The Malaria Indicator Survey (MIS), was carried out in 2015 by the National Malaria Control Program, Ministry of Health, Kenya [7]; the sampling design included responses from individuals in 6481 households. In each household, women (15–49 years) were eligible for interview, and children (6 months to 14 years) were eligible for anemia and malaria testing [7]. This MIS included questions related to the household environment, including the source of drinking water, building characteristics, and the number of rooms used for sleeping. These measures correspond to the UN Sustainable Development Goal 6 (ensure access to water and sanitation for all). Household possessions includes furniture, electronics, and livestock, and provide data on wealth, access to information, and wellbeing. Household size, composition, and residence are also included. We tabulated relevant behavioral survey responses from this MIS survey result for a more detailed analysis. We linked MIS data to relevant county data for 2015. We then coded the survey responses relating to the size of the household, number of children, use of mosquito nets, and other relevant variables [57]. This dataset adds behavioral attributes used in the multivariate Geary C analysis (See Section 3.3).

#### 2.1.3. Data Availability

The DHS and MIS databases are publicly available at https://dhsprogram.com/. We created a spatial database at two spatial resolutions—DHS cluster level as well as county levels by inter-linking the DHS geospatial covariates [32] to DHS survey cluster locations allowing for more advanced spatial analysis of malaria.

### 2.2. Methods

Since the nineties, there has been much focus on the local measure of spatial autocorrelation and spatial heterogeneity to explore spatial randomness. Local indicators of spatial association (LISA) proposed by Anselin [58] based on global Moran’s I and Getis-Ord G [59,60] are now established in the field of spatial analysis as de facto standards for testing for spatial autocorrelation with considerable attention paid to statistical inferential testing and spatial weight matrix [61,62]. They are implemented into many packages, including ArcGIS and R [63,64]. The issue of spatial neighbors is critical in testing for local spatial autocorrelation. A value is calculated for each location that can be interpolated to better visualize patterns or processes in different counties of the country.

The most commonly used measure of spatial autocorrelation is Moran’s I, which uses a continuous variable, such as incidence of malaria. Spatial autocorrelation is estimated between observations of the incidence of malaria at a county in Kenya and the “spatial lag” of this location formed by averaging malaria incidence rate per 1000 of the neighboring counties [58]. Lag is based on geographical distance between an origin location and its neighbors. For Moran’s I, the cross-product is based on the deviations from the mean for the two location values (neighboring points). Values of Moran’s I range from −1 to +1, where negative values indicate negative spatial autocorrelation, and positive values indicate positive spatial autocorrelation. We used a procedure called row standardization in ArcGIS which can correct for potential bias in the sampling or the imposed aggregation scheme of the DHS. The LISA [58] measure allows the computation of a county’s similarity with its neighbor counties as well as to test the significance for each location. We also use the measure of the incidence of malaria in each location (county or cluster) and estimate spatial autocorrelation considering the spatial neighborhood selected by the user (using buffer or immediate neighbors).

The Getis-Ord G statistic is calculated by comparing the sum of an incidence (of malaria) at a point and its nearest neighbors to the sum of all points in a given study area (Kenya). G is high when there is a high value of incidence at a location surrounded by neighbors with high-value incidences; G is low when there is a low value of incidence at a location surrounded by neighbors with low-value incidences. The Getis-Ord G estimates statistical significance by calculating z-scores and *p*-values of high or low values clusters [59,60]. The Getis-Ord G denotes whether there are clusters of high/low values of malaria incidence rate per 1000 in Kenya; whereas, the Moran’s I measure indicates if there is an overall clustering of malaria incidence rate per 1000.

To adequately address the spatial heterogeneity at a local scale, we performed three types of spatial analysis. The first objective of our study is to conduct spatial hot spot analysis using two measures: local indicators of spatial autocorrelation (LISA) [58] and Getis-Ord G statistic [59]. The second objective of this paper is to determine the effectiveness of utilizing local spatial variations in environmental and social data to uncover the relationships between malaria incidence rate per 1000 and environmental and social factors based on four malaria datasets assembled using a variety of sources provided by DHS for four time-periods—2000, 2005, 2010, and 2015. Geographically weighted regression (GWR) [65,66] analyzed the spatial patterns of relationships between malaria incidence rate per 1000 and environmental and social factors at the county scale across Kenya. The GWR used in this study is a multivariate model. The focus of the GWR is to detect and account for spatial non-stationarity in variable relationships in the regression model. GWR is a spatially localized model since it assumes that relationships between regression variables may vary over space.

The third objective of this paper is to examine two vulnerable segments of the population (pregnant women and children under 5) by focusing on the spatial variation in the household social and behavioral survey responses related to risk and prevention measures. Survey responses represent answers to “yes” or “no” questions or categories or levels of choice. The question “Has mosquito bed net for sleeping” has two responses Yes or No that were coded numerically as 1 or 0. Similarly, the responses to questions “Given away a mosquito net”. The response to the question “Type of residence” includes two responses, “Urban” or “Rural” coded as 1 or 0. The question “Importance of having children sleep under a treated net” has four responses—“Not important at all”, “A little important”, “Very important”, and” Extremely important” that are coded as 0, 0.333, 0.666, and 1 respectively. Numerical responses for all sampled household were aggregated for each DHS cluster, weighted by cluster sample weight (provided by DHS), prior to aggregation to the county level. We analyzed the relationship between 14 survey variables (see Table 1) using principal component analysis (PCA) analysis. PCA analysis used SVD (singular value decomposition) and standardized transformation in GEODA [67]. Efficient algorithms exist to calculate the SVD; computing the SVD is now the standard way to calculate PCA from a data matrix implemented in GEODA.

We attempted to analyze local spatial autocorrelation in a multivariate context with the PCA data using Geary’s C [67]. Anselin [67,68] extends the application of the local Geary C statistic to a multivariate context based on PCA components, where statistical inference is estimated using a conditional permutation approach. Geary’s C calculation is similar to Moran’s I. While in Moran’s I, the cross product is based on the deviations from the mean for the two location values, for Geary C, the cross-product uses the actual values themselves at each location. The interpretation of the two measures is different; Geary’s C varies on a scale from 0 to 2, where 0 indicates perfect positive autocorrelation or clustered pattern, while 1 indicates no autocorrelation or random pattern, and 2 indicates perfect negative autocorrelation or dispersed pattern. For Geary C analysis, we used configuration of neighboring counties based on Queen’s contiguity which defines neighbors as counties sharing a common edge or a common boundary with the origin county.

## 3. Results

### 3.1. Examining Local Spatial Autocorrelation of Malaria

**Figure 3:**
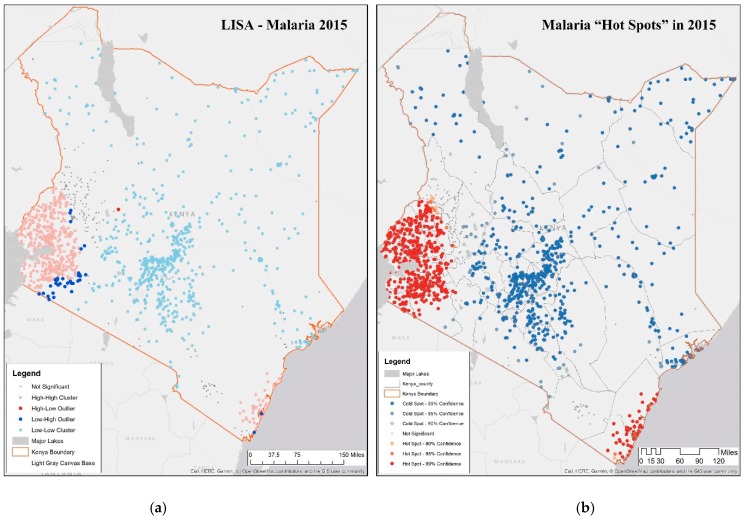
LISA and Getis-Ord G statistics characterizing local spatial autocorrelation in malaria incidence rate per 1000 in Kenya in 2015. (**a**) LISA hot spots measure showing location high-high clusters around Lake Victoria in the west and eastern sea coast; (**b**) Getis-Ord G showing three levels of statistical significance in hot and cold spots. (See Supplementary Figures S1–S6 for results in 2000, 2005, 2010).

We explored the spatial autocorrelation using the measures listed above in the four time-periods for Kenya. To estimate the spatial clustering of malaria incidence rate per 1000 from the DHS covariate database (estimated over 1000 population) across counties, LISA and Getis-Ord G were deployed to measure the extent of spatial autocorrelation among the neighboring counties. There is significant spatial autocorrelation in malaria incidence rate per 1000 in all years, indicating spatial clusters [58]. LISA helps in characterizing five possible scenarios in each time-period shown in Figure 3a.

1. A cluster with high values of malaria incidence rate per 1000 (high-high or hot spot) in all years of observation show that the Lake Victoria region is a noticeable hot spot; both rainfall and proximity to water are significantly higher than other regions of the country. A second smaller hot spot cluster is around Mombasa, on the east coast; these two hot spots appear across all four years with minor differences along the boundary regions, where there are some outliers. These two regions in 2015 coincide with DHS malaria zones called the coastal endemic and lake endemic regions, as shown in Figure 1b.
2. A cluster with low values of malaria incidence rate per 1000 (low-low or cold spot) indicating low or no disease, located in the northern part of the country as well as around Nairobi; these regions are in the DHS semi-arid and seasonal risk areas. The only difference across the four periods is the appearance of some outliers on the outer edges of Lake Victoria endemic region indicating a “boundary” effect as locations at the boundary of 2 zones flip from being insignificant to some level of significance in some time-periods.
3. An outlier of high value of malaria incidence rate per 1000 surrounded by a low value (high-low), is found in Baringo, which is in the highland epidemic region, with semi-arid, seasonal risk; this outlier is visible in 2015, as shown in Figure 3a. Such locations need scrutiny since people may be unprepared while at risk.
4. An outlier of low value of malaria incidence rate per 1000 surrounded by a high value (low-high), is prevalent in Nakuru, lying in a low risk area neighboring Lake endemic malaria zone in the southwest. This pattern persists through all the time-periods.
5. Non-significant values encompass all areas in which there were no significant associations, and are found in Trans Nzoia and Uasin Gishu, classified as being in the highland epidemic [49].

In summary, general patterns of hot and cold spots appear in the same regions across the four years of observation. Figure 3a shows LISA for 2015, similar to LISA in earlier years, indicating that there are consistent hot spots corresponding to DHS endemic zones. Efforts for malaria eradication and prevention should be directed to regions that show consistent patterns of risk as well as a potential risk due to climate change in transitional zones sharing boundaries with lake and coastal endemic regions, colored in red, purple, and yellow in Figure 1b.

Positive and significant values of the Getis-Ord G indicate a cold spot, or cluster of low values [59,60]. In Figure 3b, Getis-Ord G statistics are provided at 3 levels of confidence, 90%, 95%, and 99%. These results across the four time-periods mirror the LISA results with correspondence in the hot and cold spot clusters as well as insignificant clusters in Trans Nzoia, Uasin Gishu, and Taita Taveta in 2015.

As such, there is considerable overlap between LISA and Getis-Ord G since both agree on the hot and cold spots. The only difference is the low-high and high-low outliers in the transitional buffer highlighted in the LISA analysis. Both analyses suggest that malaria eradication efforts should be directed at lake and coastal endemic counties that are significant hot spots in both analyses as well as the centers of expanding population in urban and semi-urban areas.

### 3.2. Spatial Determinants of Malaria

The next analysis examines the spatial determinants underlying these clusters. We can now analyze these spatial patterns of malaria incidence rate per 1000 through time using the determinants—proximity to water, rainfall, population, and vegetation. These factors were found to be relevant (step-wise regression), and consistently available for all years of study.

Hot spot analysis is run on OLS residuals to check for spatial pattern of over and under predictions that can provide clues about missing critical variables from the model. Table 2 shows Moran’s I values of spatial autocorrelation of OLS residuals of malaria incidence rate per 1000 from the 2000–2015 period (see Table 2 - row 1). The highest value of Moran’s I is in 2015, while values are significant for clustering in all four periods of observation (last row in Table 2).

**Figure 4:**
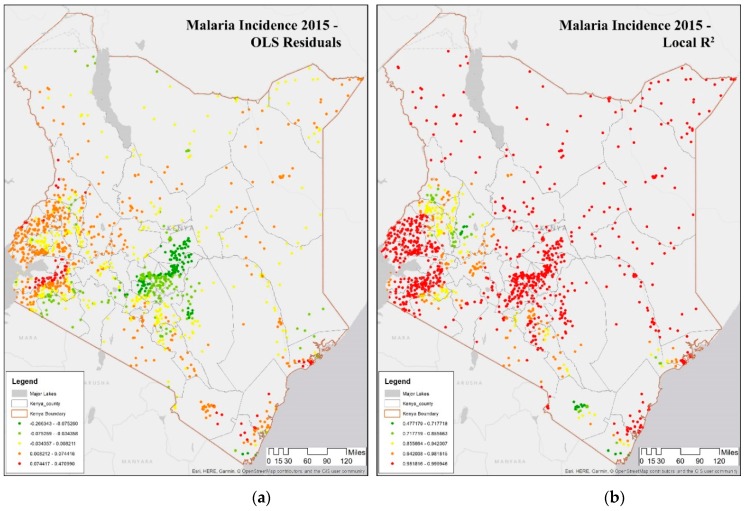
Ordinary Least Squares (OLS) and Geographically Weighted Regression (GWR) analysis of malaria incidence rate per 1000 in 2015. (**a**) Map of OLS residuals show the over and under-prediction of OLS results suggesting spatial variation in the determinants of malaria; (**b**) R^2^ values of malaria incidence rate per 1000 in 2015 using GWR. Higher R^2^ are shown in red while lower values are shown in green.

In Figure 4a, OLS residuals are negative (green), suggesting over-prediction in malaria incidence rate per 1000 around Nairobi, while there is some under-prediction around Lake Victoria and the east coast. The spatial autocorrelation in OLS residuals (red and green clusters) is due to nonstationary spatial processes. GWR is useful in analyzing local spatial heterogeneity in malaria incidence rate per 1000 represented by R^2^ values shown in Figure 4b. Higher R^2^ in spatial regression (in red) in Figure 4b occurs around three regions—Lake Victoria, Nairobi, and the east coast. There are lower R^2^ values in counties near (in green) Lake Victoria and Taita Taveta in the south.

**Figure 5:**
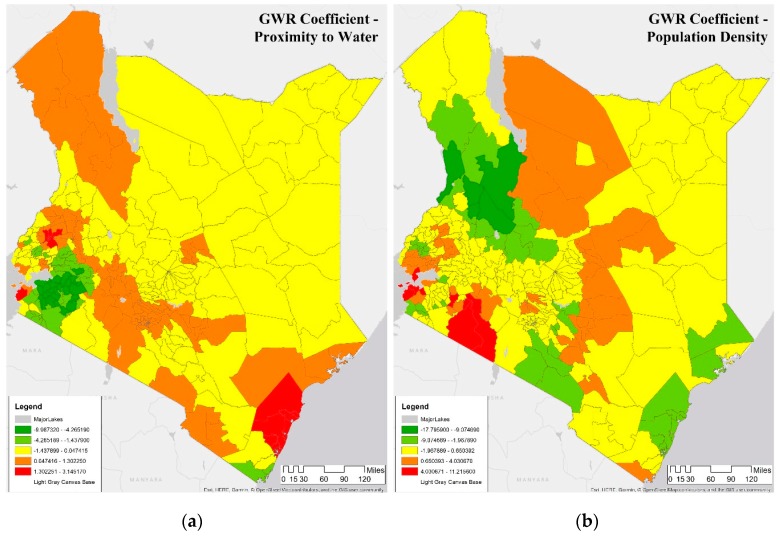

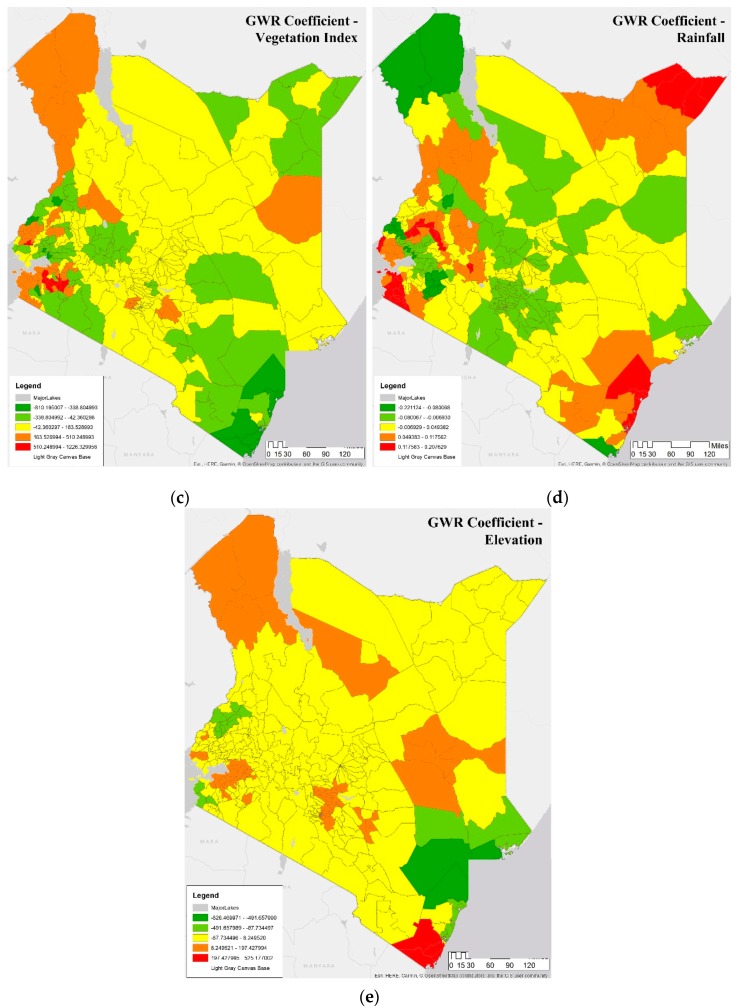
Five Geographically Weighted Regression (GWR) coefficients of malaria incidence in counties of Kenya in 2015. (**a**) Map of GWR coefficients of proximity to water showing negative values in the west and more positive values around Nairobi. (**b**) Map of GWR coefficient of population density showing differences in the impact in the west. (**c**) Map of GWR coefficients of vegetation showing differences around Lake Victoria in the west. (**d**) Map of GWR coefficients of rainfall shows east to west spatial differentiation. (**e**) Map of GWR coefficients of elevation shows a correlation to vegetation and rainfall. All coefficients results are significant with *p* < 0.001. (See Supplementary Figures S7–S21 for results in 2000, 2005, 2010).

Figure 5 shows the GWR coefficient values estimated (using spatial Kriging technique) over the counties using the original DHS covariates and malaria incidence rate per 1000 data for 2015. The following discussion of determinants covers the four observation periods. The coefficient value of proximity to water is shown in Figure 5a; the coefficient value of proximity to water is negative in explaining malaria incidence rate per 1000, i.e., as proximity to water decreases, the incidence of malaria increases. Exceptions are the counties of Siaya, Trans Nzoia, and Uasin Gishu (northwest), shown in Figure 5a, where coefficients are large and positive. Also, this coefficient value is large and positive around Mombasa for 2000, 2005, and 2015. For 2010, the coefficient is not significant. Similarly, this coefficient is positive around Murang’a and negative around Kirinyaga for 2000 and 2015. (Both counties are around Nairobi). Kirinyaga has a positive coefficient in 2005, suggesting some change. Similar to Mombasa, the coefficient was not significant around the Nairobi region in 2010.

Figure 5b shows the significance of the population density determinant in 2015. Population density is more crucial for the northern shore than the southern shore of Lake Victoria in 2000, 2005, and 2015. However, the population becomes paramount for the southern shore in 2015, due to higher fertility, and increased life expectancy in the southern region of the Nyanza province in Kenya [46]. Around Nairobi, the significance of population determinant varies, perhaps reflecting population growth from 2000 to 2015. Nairobi is estimated to have an annual population growth rate of 4% and contains 25% of the country’s population. Overall, population size is not significant in explaining malaria incidence rate per 1000 around Mombasa.

Vegetation coefficients, shown in Figure 5c for 2015, are significant in determining malaria incidence rate per 1000 and are positive in the southern shore of Lake Victoria, and are negative on the northern shore of Lake Victoria. Prior studies highlight the complex nature of malaria vector breeding in the lake habitats and describe the role of short and tall grass as well as water hyacinths in the lake [47,48]. The vegetation index coefficient was positive for Mombasa in 2000. However, the value became negative after 2000, perhaps reflecting the removal of mangrove forest in this coastal region [69]. Nairobi and surrounding areas such as Nyeri, which surrounds Mt. Kenya while having higher vegetation cover, seem to have a positive relationship with malaria and maybe indicative of climatic effects of malaria in Kenyan highlands as documented in studies showing of a resurgence of malaria in highland areas of Kenya [70].

In Figure 5d, rainfall coefficients are higher and are significant in determining malaria incidence rate per 1000 around Mombasa from 2000 to 2005, south of Lake Victoria from 2010 to 2015, while rainfall has smaller coefficients around Nairobi from 2005 to 2010. The ENSO event of 2015 is perhaps more significant in driving up malaria in the southern region of Lake Victoria, as noted in prior research [47,48]. There are some changes in the rainfall coefficient over the time-period, shown in Figure 5d, around northern Lake Victoria. Figure 5e shows elevation coefficients. Elevation in Kenya varies from sea level to more than 4000 m. The most significant positive elevation coefficients are in provinces that exhibit a range of elevation such as in the southeast in the county of Taita Taveta with an elevation ranging from 500 to 2000 m, (located northwest of Mombasa and southeast of Nairobi). Other counties showing positive elevation coefficients are in the counties of Turkana and Marsabit in the northwest. With climate change, highland regions of Kenya are experiencing warmer temperatures and have seen an increase in malaria [69,71,72,73]. Malaria is infiltrating new areas, where the population had little exposure to the disease and no natural immunity [72].

**Figure 6:**
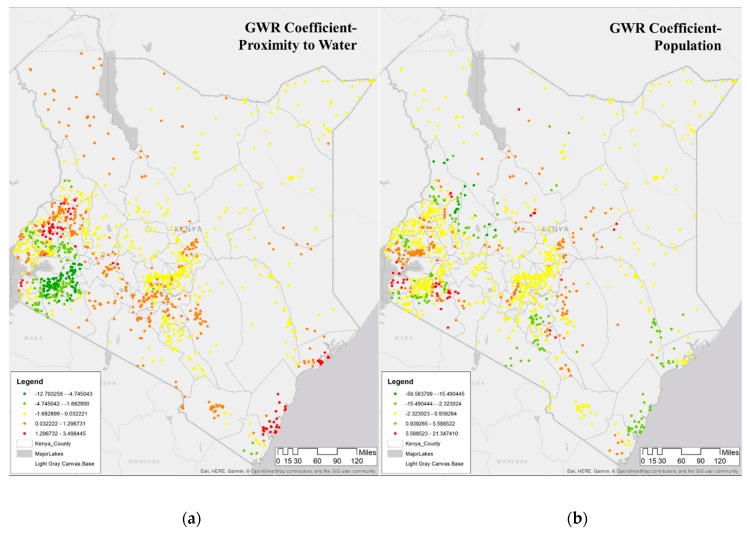
GWR Coefficients of malaria incidence rate per 1000 in the DHS clusters surveyed in 2015, estimated from original data. (**a**) Map of GWR coefficient of proximity to water showing negative values in the west and more positive values in the north and some in the east. (**b**) Map of GWR coefficient of population density showing differences in the impact around major cities. (See Supplementary Figures S22–S41 for GWR coefficients for all variables for 2000, 2005, 2010, 2015).

Figure 6 shows the coefficients of two covariates in the DHS clusters in 2015, whose interpolated surface is shown in Figure 5. Figure 6a shows proximity to water, while Figure 6b shows population density. For the majority of the Lake Victoria region (southeast), the coefficient value of proximity to water is negative, i.e., as the proximity to water decreases, the incidence of malaria increases, as seen in Kisumu, Vihiga, Siaya, and Homa Bay. To summarize, the proximity to water is a major determinant of malaria in this region, and therefore, a public health warning needs to focus on this determinant. Some counties in this lake endemic regions are exceptions such as counties of West Pokot, Trans Nzoia, and Uasin Gishu (northwest) where the value of the determinant is significant indicating that some other factor may be relevant in this region. Note that the GWR model is predicting well in the endemic malaria region around Lake Victoria, as seen in Figure 2b and Figure 4b.

To summarize, Figure 5 and Figure 6 shows the spatial heterogeneity in the coefficients of determinants in characterizing malaria in 2015. Rainfall and proximity to water and vegetation are associated with increased malaria risk in many DHS locations. The population is significantly higher in Nairobi, which is a malaria cold spot since Nairobi is in the highland region with a cooler climate and is better protected. These results confirm that proximity to water is a crucial determinant of the malaria incidence rate per 1000. Additionally, the results also present more nuanced outcomes in the malaria incidence rate per 1000 in Kenya. First, Nairobi and surrounding areas such as Nyeri, which surrounds Mt. Kenya while having higher vegetation cover, seem to have a positive relationship with malaria and maybe indicative of climatic effects of malaria in Kenyan highlands as documented in studies showing of a resurgence of malaria in highland areas of Kenya [70,71,72]. Second, changes in vegetation cover combined with rainfall may be resulting in higher malaria cases [69,73]. Finally, there seems to be an expansion of malaria endemicity to counties such as West Pokot, Trans Nzoia, and Uasin Gishu.

### 3.3. Spatial Analysis of Social, Demographics, Housing, and Behavior Characteristics of the Vulnerable Population

Tabulated MIS survey results described in Section 2.1.2 were analyzed PCA; we investigate the correlation and significance of factors related to 14 variables shown in Table 1. All variables were continuous values except four that were categorical levels [7]. They include (i) Has mosquito bed net for sleeping; (ii) Given away a mosquito net; (iii) Type of place of residence; and (iv) Importance of having children sleep under a treated net.

The first two PCA components account for 88% of the total variance which denote the significance of the size of the household, the number of children, and the number of nets. Table 2 shows the PCA 1 and 2 loadings which account for the variance. PCA 1 relates to size of the household, as the variables with the highest scores are “# Of Household Members”, “# Of Women”, “# of Mosquito Bed Nets”, “# Of Children Under 5”, and “# Of Children Under 5 Slept under Net Last Night”, are correlated with household size. Loadings are positive indicating that all the values and all the variables in a component are positively correlated with each other. On the other hand, PCA 2, representing the dimension characterizing children, has negative loadings. PCA 2 captures the inverse relationship between “Type of Place of Residence” and “Imp. of Having Children Sleep under a Tr. Net” and variables such as “# Of Children Under 5 Had Fever”, “# Children Under 5 Received Treatment”, and “# Of Children under 5” [49].

**Figure 7:**
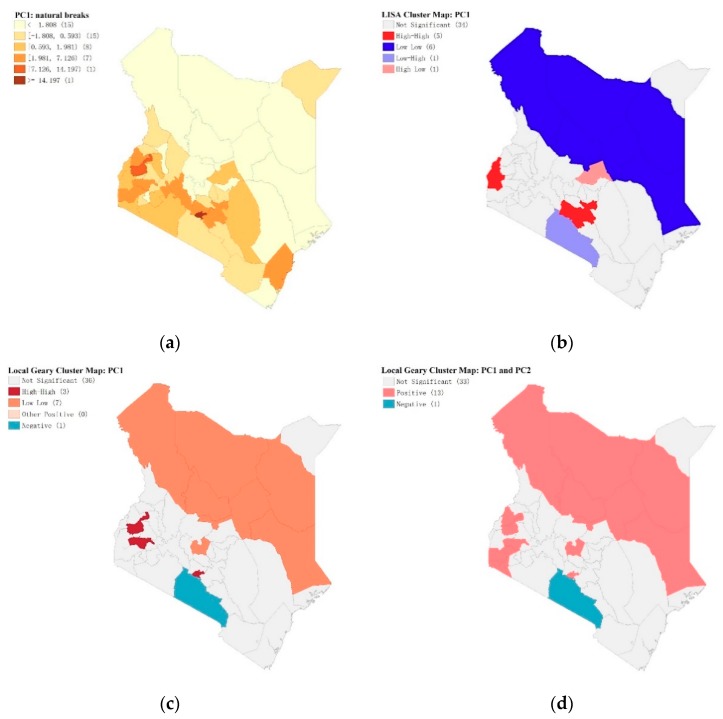
C and Moran’s I statistics (**a**) Map of PCI natural breaks showing high values in the two endemic areas. (**b**) Local Moran’s clusters, PC1 showing low-low clusters of survey responses. (**c**) Local Geary’s C clusters, PC1. (**d**) Bivariate local Geary, PC1, and PC2; notice negative cluster south of Nairobi in Kajiado county. (See Supplementary Figures S42 and S43 for Map of PC2 natural breaks and LISA and Local Geary’s C Clusters).

We employ local Geary’s C in a multivariate setting, as described by Anselin [68] to compare geographical neighbors with neighbors in multi-attribute space. We map the first PCA in Figure 7a using natural breaks to show distribution. These figures demonstrate that social and behavioral responses linked with the household size of PCA 1 are significant in the lake endemic, and the coastal endemic regions as well as in the transitional zones at the boundaries. Figure 7b shows the PCA 1 hotspot map using Moran’s I; counties of Nairobi and Kakamega show high-low trends (high PCA 1 surrounded by low PCA 1), while northern counties are cold spots (low values of PCA 1). Figure 7c shows the same map using Geary’s C. Figure 7b,c shows the similarities in clusters using the two methods covering northern Kenya (except Mandera county). Figure 7c shows that Geary’s C highlights one low areas (in blue) located in Kajiado county, on the southern boundary. Figure 7d displays the bivariate local Geary clusters applied to the first two principal components [67,68]; we estimate the false discovery rate (FDR) at 99% confidence interval (based on 99,999 permutations) [68] for the bivariate local Geary clusters depicted on Figure 7d. Compared to the individual local Geary cluster maps for each variable, we obtain a map of counties for both positive and negative bivariate clusters. This indicates that Lake bivariate cluster is positive while the southern province of Kajiado is negative, suggesting a differential approach to prevention [70]. The Lake region and Nairobi have low-low clusters in both LISA and Geary’s C maps for PC2 (see Supplementary Material shows PC2—natural breaks, LISA cluster map, local Geary’s C map). Malaria efforts in the negative cluster may need more monitoring and vigilance of the underlying determinants, including rainfall and vegetation [71].

## 4. Discussion

The DHS program provides the best available data for malaria given the paucity of real data concerning vulnerable populations on malaria across Kenya. The malaria surveys in Kenya provide the best available spatio-temporal datasets for characterizing spatial distribution of malaria at a nationally representative level. The hot spot analysis using three different measures indicate the clustered pattern of malaria incidence rate per 1000 in Kenya. There are significant hot spots or clusters of the disease incidence around Lake Victoria, Kisumu and Kakamega, and Mombasa. The rest of the country, including the densely populated capital Nairobi and the less populated northern counties such as Mandeara, Marsabit, Samburu, and Wajir, are characterized as cold spots of low incidence of malaria. Stable patterns in malaria incidence rate per 1000 occur on the eastern coast around Mombasa and the western counties around Lake Victoria in the time-period. 

Knowledge of “hotspot” areas of high malaria incidence rate per 1000 is critical in mobilizing preventive interventions in resource-poor areas, mainly if the hotspot areas can be predicted using determinants such as rainfall or vegetation. The GWR analysis shows that the determinants of malaria incidence rate per 1000 in Kenya are influenced by rainfall, vegetation, and proximity to water similar to prior research using other methods [69,70,71,72,73]. We analyzed the impact of wealth status in 2015. This is another relevant variable that may be related to individuals seeking preventive treatment. Our analysis also suggests that transitional areas around endemic areas need to be more vigilant in terms of malaria prevention since they are potentially at risk. Malaria in urban areas has seen effective vector control, but the increasing urban sprawl and expansion into semi-urban areas now pose new challenges in vector control and dissemination of public warnings [74,75]. Regional pattern and clustering of indicators used in the study suggest a need to continue and bolster county level and locally focused prevention and eradication programs. Mapping of the variation in malaria can help in improving programs in terms of the allocation of limited resources to those regions with highest needs of healthcare. The meteorological factors including rainfall and proximity to water are found to be positively associated with malaria incidence rate per 1000 and prevalence. Finally, prevention and control activities need to be integrated with agriculture and irrigation schemes.

We highlight the potential limitations of the MIS & DHS data, as they are all estimated and not actual patient data; these estimations may be less accurate than actual patient data. Our future efforts will focus on actual patient-level admissions and treatment data from hospitals and clinics that can be georeferenced and analyzed using the methods outlined in this paper. Such georeferenced data may shed light on malaria attributed to seasonal/holiday travel to malaria hotspots, temporary migration (high school students headed to boarding schools from malaria hotspots), and changes in rainfall or climate leading to higher altitudes experiencing malaria for the first time. Additionally, the public in Kenya routinely self-medicate for both malaria treatment and prophylaxis by purchasing medications in drug stores without a prescription. While the current survey does ask questions on antimalarial medication use, it does not explicitly address the question on self-medication. As such, spatial analysis of behavioral patterns and responses around travel, migration, and self-medication vis-a-viz malaria would be a critical addition to the literature and in the analysis of spatial patterns of malaria incidence. In a future paper, we plan to combine our spatial model of determinants with state-of-the-art numerical climate models to predict adverse malaria events and identify those regions most likely to require intervention in a given year based on fluctuations in climate.

## 5. Conclusions

The findings of the present study are crucial for county level planning and policy making in tackling and eradicating malaria in Kenya by 2030. The present work further provides the linkages between DHS cluster level county level analysis, uncovering variables that are either negatively or positively related to malaria incidence. Finally, by integrating climatic, environmental, socio-economic status variables measured at the county level, the present paper is able to contribute in filling the research gap in identifying the importance of the contextual correlates and spatial neighborhood for malaria in Kenya. Estimating the effects of these contextual factors may help in identifying the vulnerable urban and rural local population. Our study shows the promise of using nationally representative datasets to better understand the nuances in malaria incidence and prevention.

## Figures and Tables

**Table 1:** Showing PCA results for 14 variables. The first two components explain 87% of the total variance.

Variables	Variable Loadings in 2015	
	PC1	PC2
# of Mosquito Bed nets	0.279199	0.0778586
# Of Children Under 5 Slept under Net Last Night	0.278166	−0.175717
# Of Children Slept under Net Last Night	0.237191	−0.337577
# Of Children under 5 Had Fever	0.253837	−0.337189
# Children under 5 Received Treatment	0.257043	−0.302508
# Of Children under 5	0.274868	−0.207184
# Of Household Members	0.294541	−0.012094
# Of Women	0.286635	0.194498
# Of Children	0.258989	−0.327962
# Of Pregnant Women	0.266416	0.15378
Has Mosquito Bed Net for Sleeping	0.285655	0.191331
Given Away a Mosquito Net	0.247505	0.291434
Type of Place of Residence	0.236617	0.476703
Imp. of Having Children Sleep under a Tr. Net	0.276666	0.288097
Importance of components:		
Standard deviation	3.274989	1.255193
Proportion of Variance	0.766111	0.112536
Cumulative Proportion	0.766111	0.878647

**Table 2:** Moran’s I on OLS residuals suggesting significant autocorrelation of residuals.

Values	2000	2005	2010	2015
Moran’s I	0.666407	0.585607	0.642730	0.698402
Expected Index:	0.000732	0.000775	0.000775	0.000775
Variance:	0.000077	0.000085	0.000085	0.000086
z-score:	75.796087	63.427044	69.677760	75.567593
*p*-value:	<0.001	<0.001	<0.001	<0.001

## Supplementary Materials

The following are available online at https://www.mdpi.com/1660-4601/16/24/5078/s1.
