# Supplementary material for: Characterizing the Spatial Determinants and Prevention of Malaria in Kenya
Source: Int J Environ Res Public Health. 2019 Dec 12;16(24):5078. doi: 10.3390/ijerph16245078 (PMC6950158; doi:10.3390/ijerph16245078)
Supplement: Supplementary file 1 [file ijerph-16-05078-s001.pdf]

Supplementary Figures

Figure S1. Map of malaria incidence hotspots, cold spots and outliers in 2000 (LISA)

Figure S2. Map of malaria incidence hotspots, cold spots and outliers in 2005 (LISA)

Figure S36. Map of malaria incidence hotspots, cold spots and outliers in 2010 (LISA)

Figure S4. Map of malaria incidence hotspots and cold spots in 2000 (Getis-Ord Gi*)

Figure S5. Map of malaria incidence hotspots and cold spots in 2005 (Getis-Ord Gi*)

Figure S6. Map of malaria incidence hotspots and cold spots in 2010 (Getis-Ord Gi*)

Figure S7. Map of GWR coefficients of proximity to water in 2000 (aggregated to county level)

Figure S8. Map of GWR coefficients of proximity to water in 2005 (aggregated to county level)

Figure S9. Map of GWR coefficients of proximity to water in 2010 (aggregated to county level)

Figure S10. Map of GWR coefficients of population density in 2000 (aggregated to county level)

Figure S11. Map of GWR coefficients of population density in 2005 (aggregated to county level)

Figure S12. Map of GWR coefficients of population density in 2010 (aggregated to county level)

Figure S13. Map of GWR coefficients of EVI (enhanced vegetation index) in 2000 (aggregated to county level)

Figure S14. Map of GWR coefficients of EVI (enhanced vegetation index) in 2005 (aggregated to county level)

Figure S15. Map of GWR coefficients of EVI (enhanced vegetation index) in 2010 (aggregated to county level)

Figure S16. Map of GWR coefficients of rainfall in 2000 (aggregated to county level)

Figure S17. Map of GWR coefficients of rainfall in 2005 (aggregated to county level)

Figure S18. Map of GWR coefficients of rainfall in 2010 (aggregated to county level)

Figure S19. Map of GWR coefficients of elevation in 2000 (aggregated to county level)

Figure S20. Map of GWR coefficients of elevation in 2005 (aggregated to county level)

Figure S21. Map of GWR coefficients of elevation in 2010 (aggregated to county level)

Figure S22. Map of GWR coefficients of proximity to water in 2000 (at DHS cluster)

Figure S23. Map of GWR coefficients of proximity to water in 2005 (at DHS cluster)

Figure S25. Map of GWR coefficients of proximity to water in 2010 (at DHS cluster)

Figure S25. Map of GWR coefficients of proximity to water in 2015 (at DHS cluster)

Figure S26. Map of GWR coefficients of population density in 2000 (at DHS cluster)

Figure S27. Map of GWR coefficients of population density in 2005 (at DHS cluster)

Figure S28. Map of GWR coefficients of population density in 2010 (at DHS cluster)

Figure S29. Map of GWR coefficients of population density in 2015 (at DHS cluster)

Figure S30. Map of GWR coefficients of EVI (enhanced vegetation index) in 2000 (at DHS cluster)

Figure S31. Map of GWR coefficients of EVI (enhanced vegetation index) in 2005 (at DHS cluster)

Figure S32. Map of GWR coefficients of EVI (enhanced vegetation index) in 2010 (at DHS cluster)

Figure S33. Map of GWR coefficients of EVI (enhanced vegetation index) in 2015 (at DHS cluster)

Figure S34. Map of GWR coefficients of rainfall in 2000 (at DHS cluster)

Figure S35. Map of GWR coefficients of rainfall in 2005 (at DHS cluster)

Figure S36. Map of GWR coefficients of rainfall in 2010 (at DHS cluster)

Figure S37. Map of GWR coefficients of rainfall in 2015 (at DHS cluster)

Figure S38. Map of GWR coefficients of elevation in 2000 (at DHS cluster)

Figure S39. Map of GWR coefficients of elevation in 2005 (at DHS cluster)

Figure S40. Map of GWR coefficients of elevation in 2010 (at DHS cluster)

Figure S41. Map of GWR coefficients of elevation in 2015 (at DHS cluster)

Figure S42. Left: Map of PC2 natural breaks; Right: Map of PC2 hotspots, cold spots and outliers (LISA)

Figure S43. Map of Local Geary's C Clusters, PC2

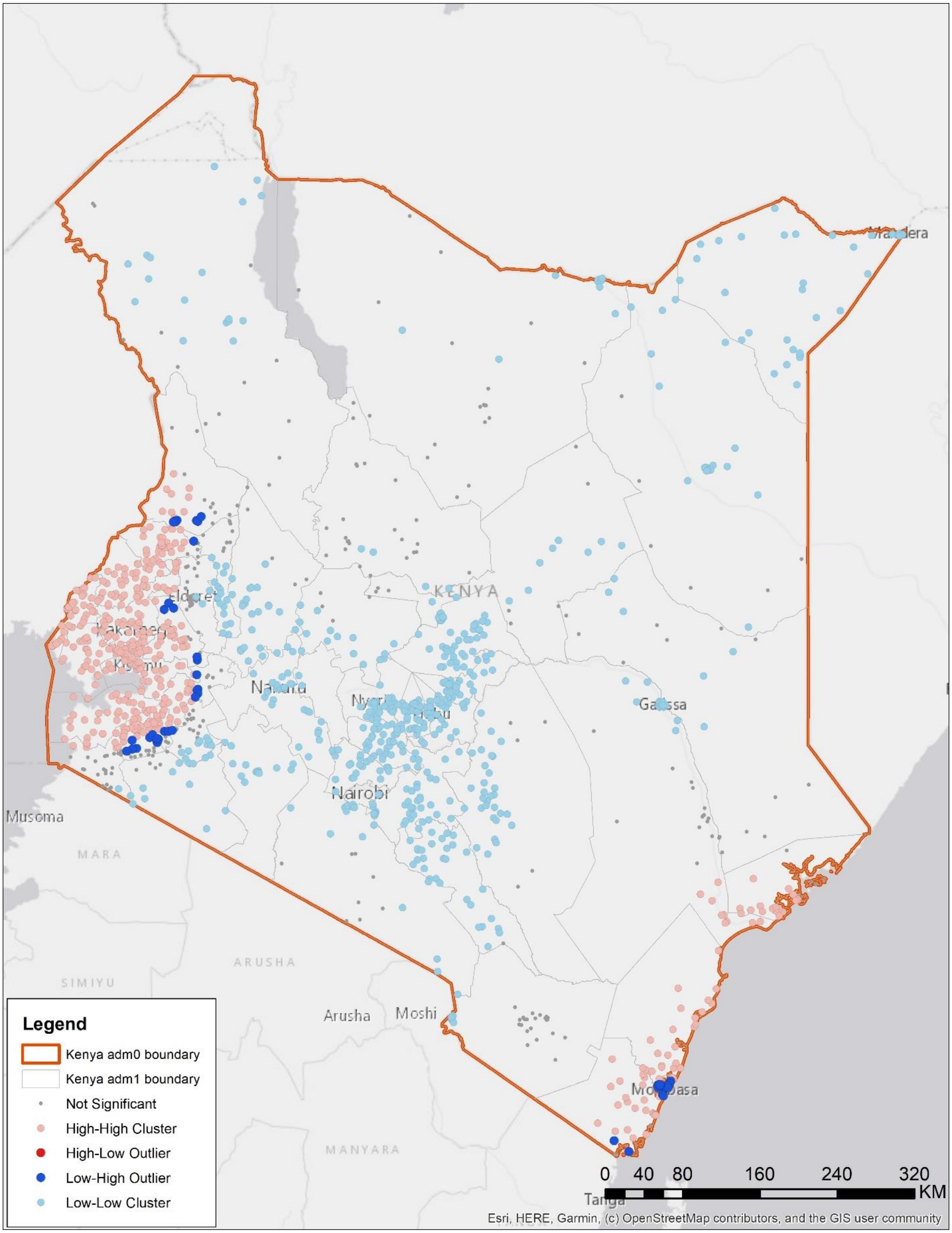

Figure S1. Map of malaria incidence hotspots, cold spots and outliers in 2000 (LISA)

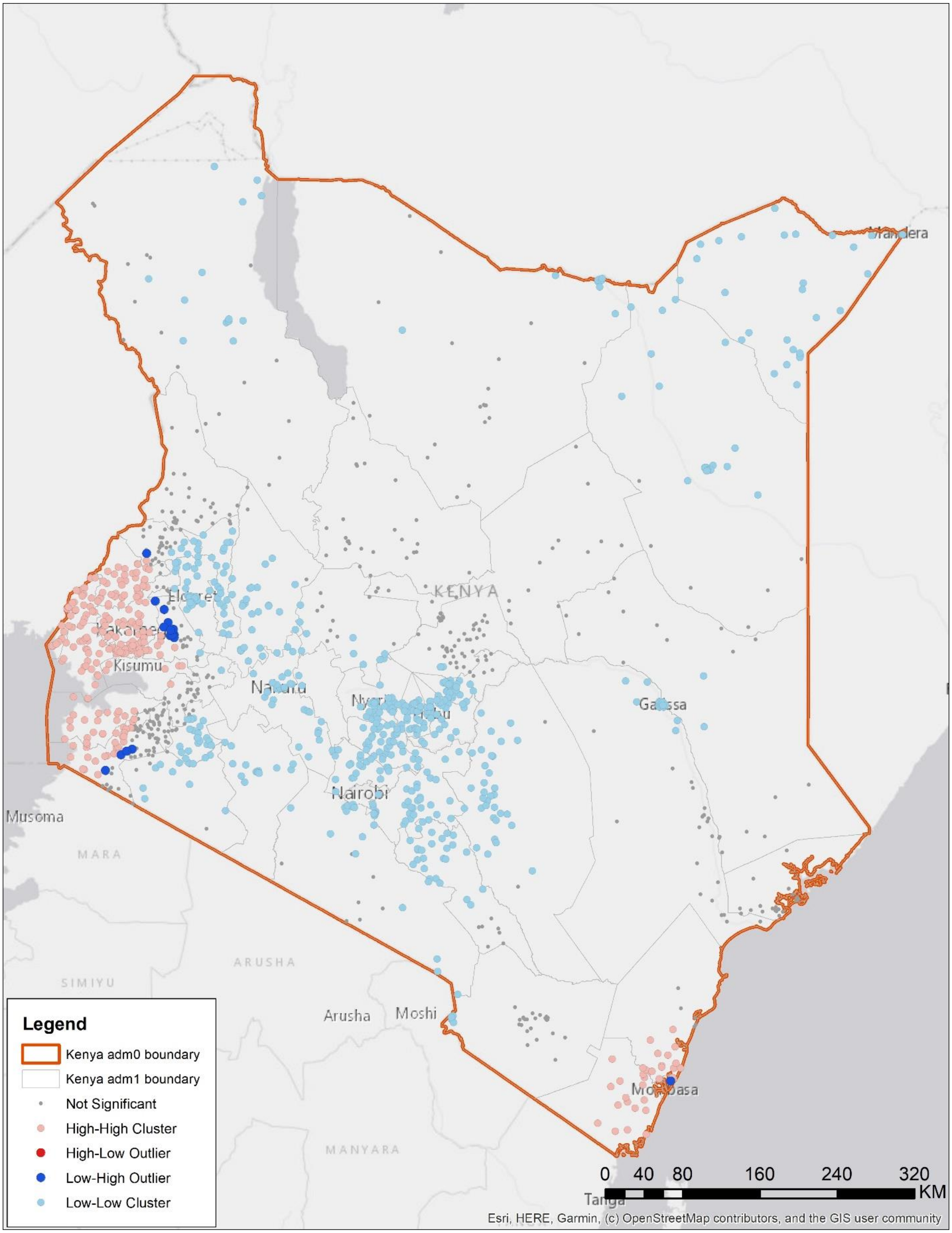

Figure S2. Map of malaria incidence hotspots, cold spots and outliers in 2005 (LISA)

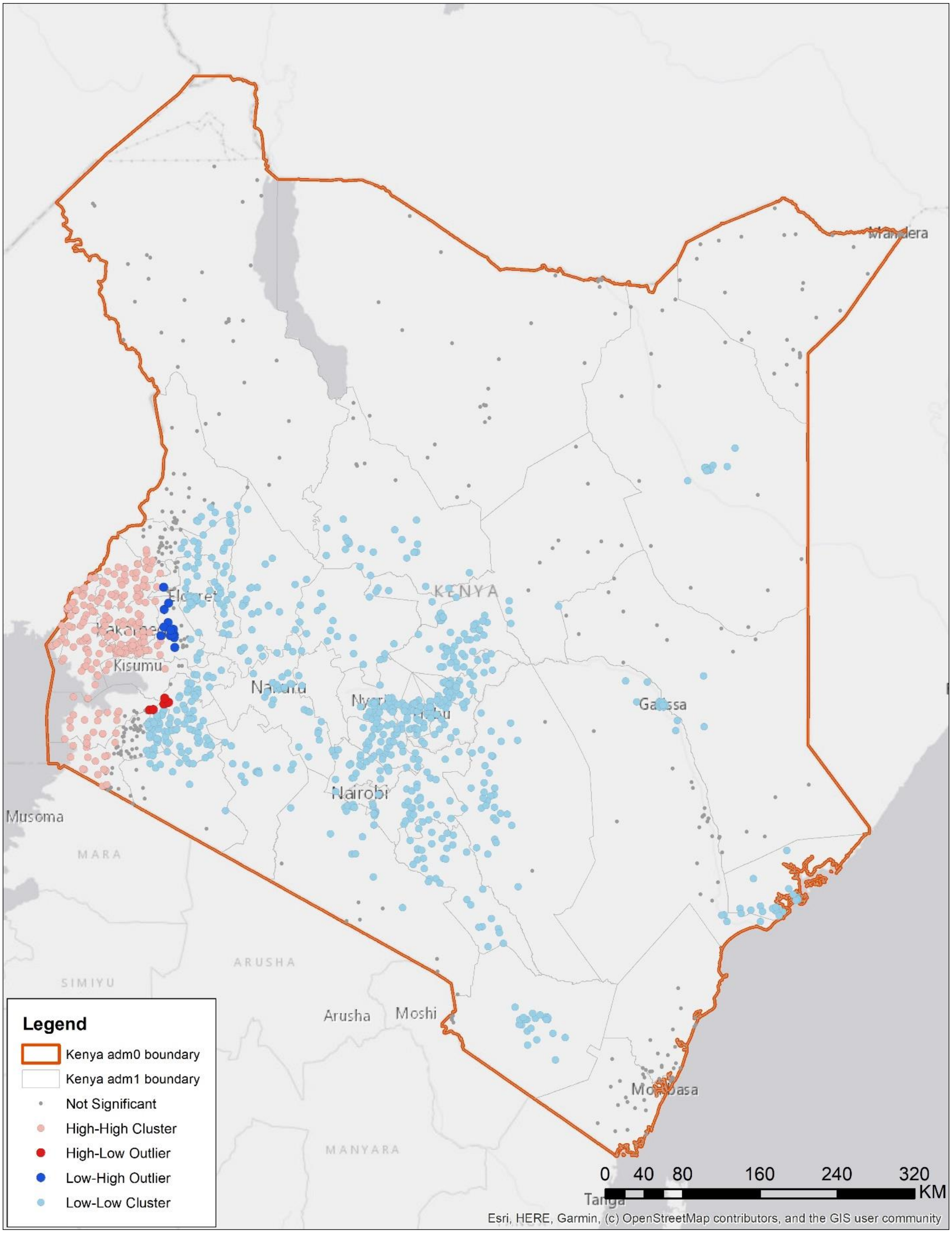

Figure S3. Map of malaria incidence hotspots, cold spots and outliers in 2010 (LISA)

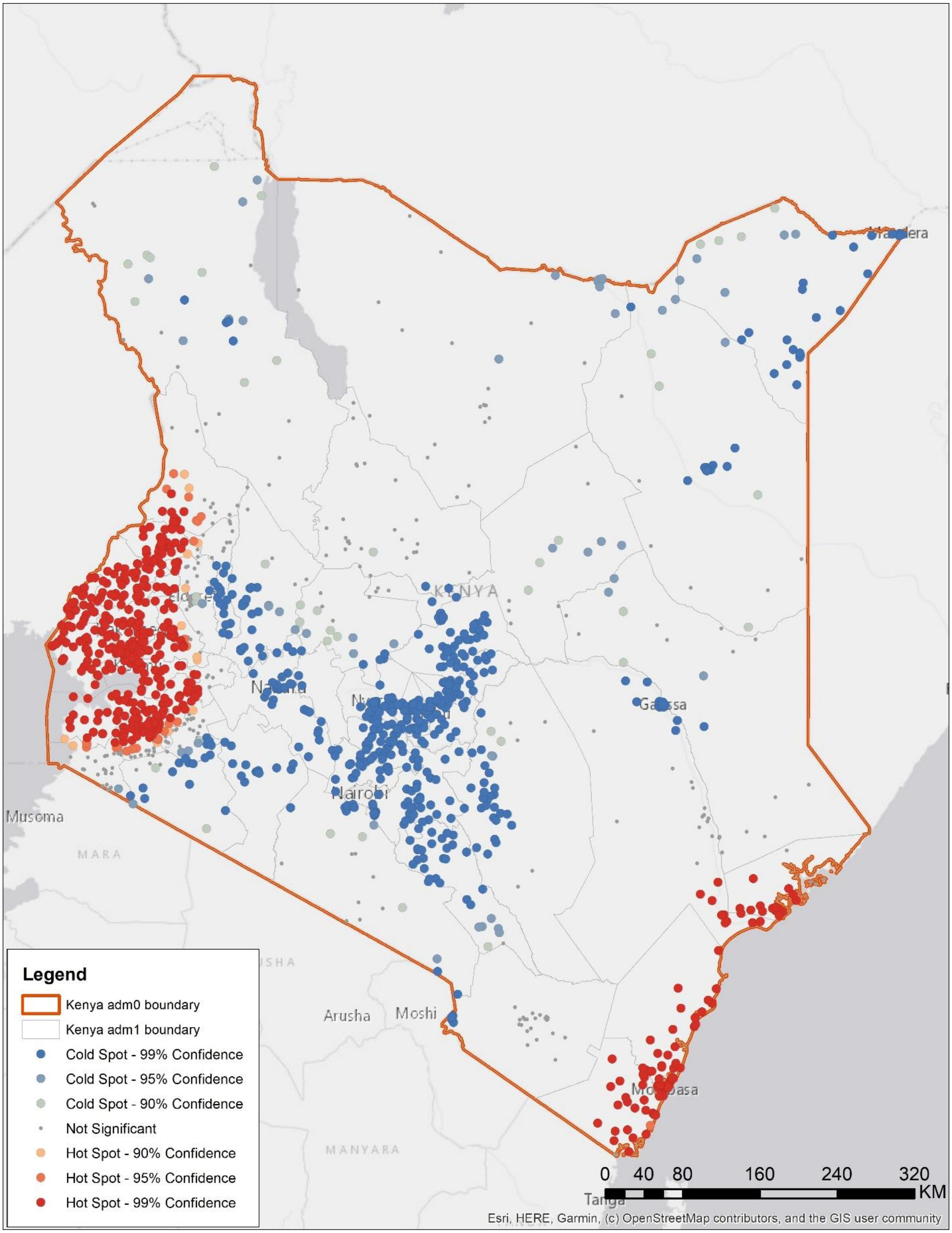

Figure S4. Map of malaria incidence hotspots and cold spots in 2000 (Getis-Ord Gi*)

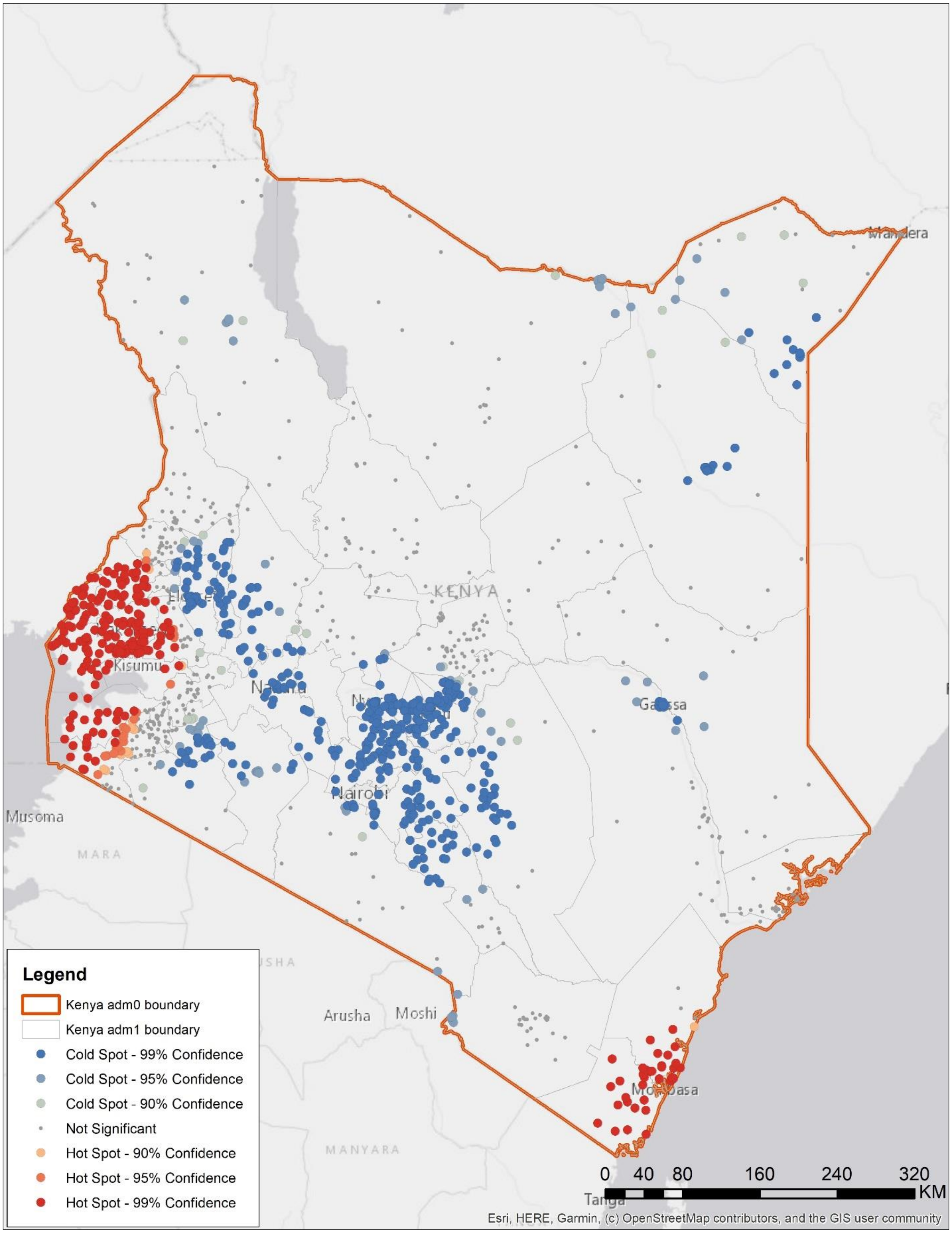

Figure S5. Map of malaria incidence hotspots and cold spots in 2005 (Getis-Ord Gi*)

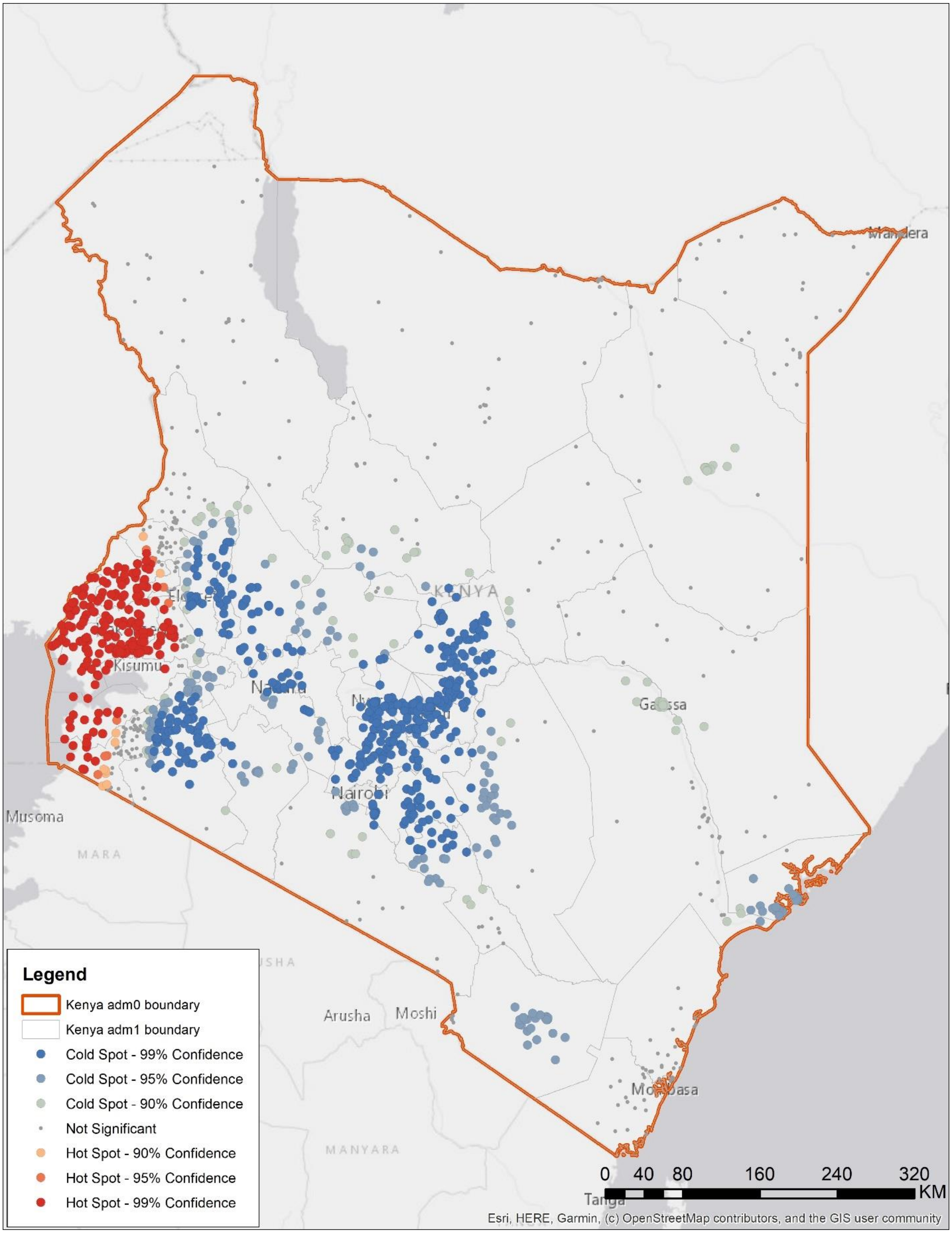

Figure S6. Map of malaria incidence hotspots and cold spots in 2010 (Getis-Ord Gi*)

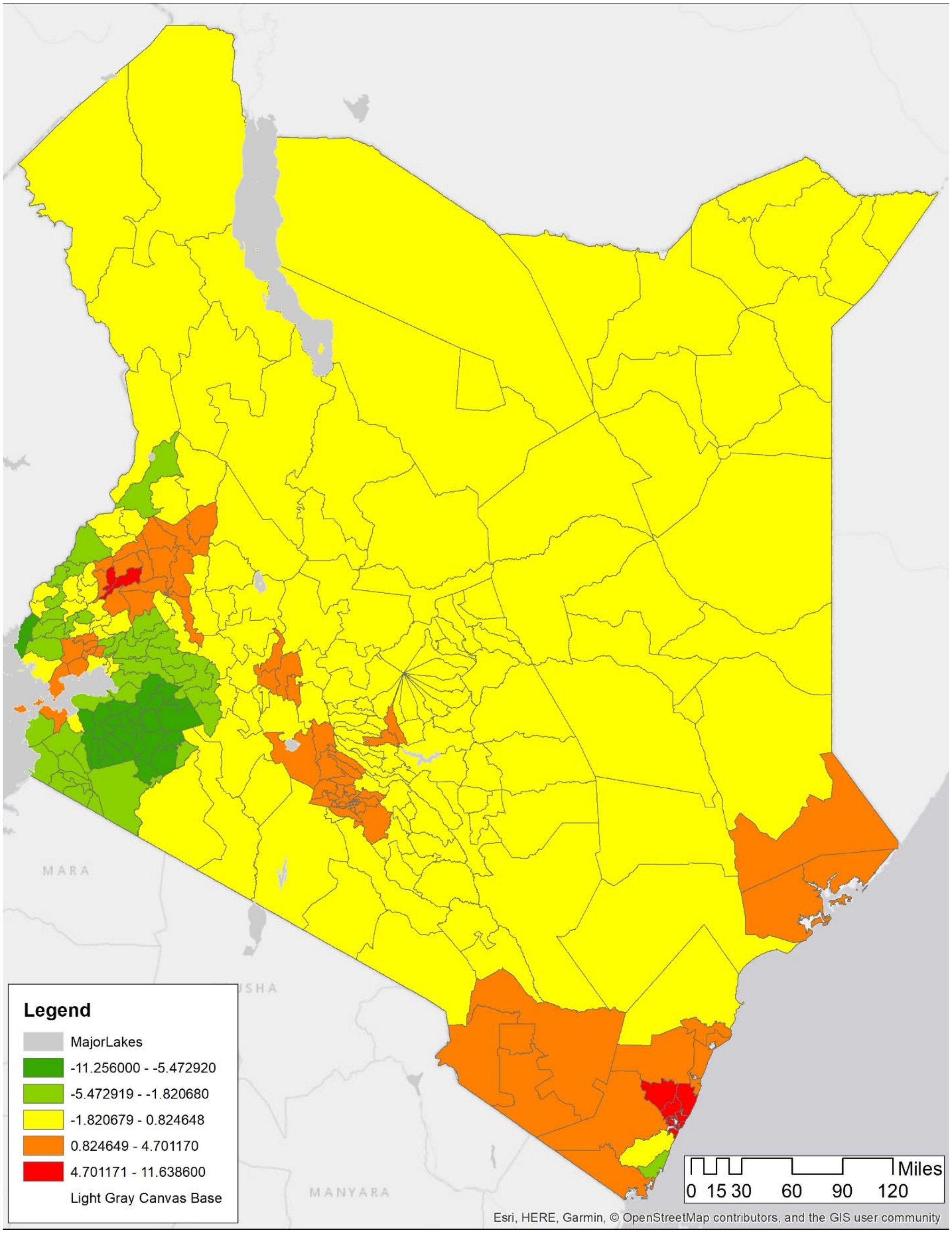

Figure S7. Map of GWR coefficients of proximity to water in 2000 (aggregated to county level)

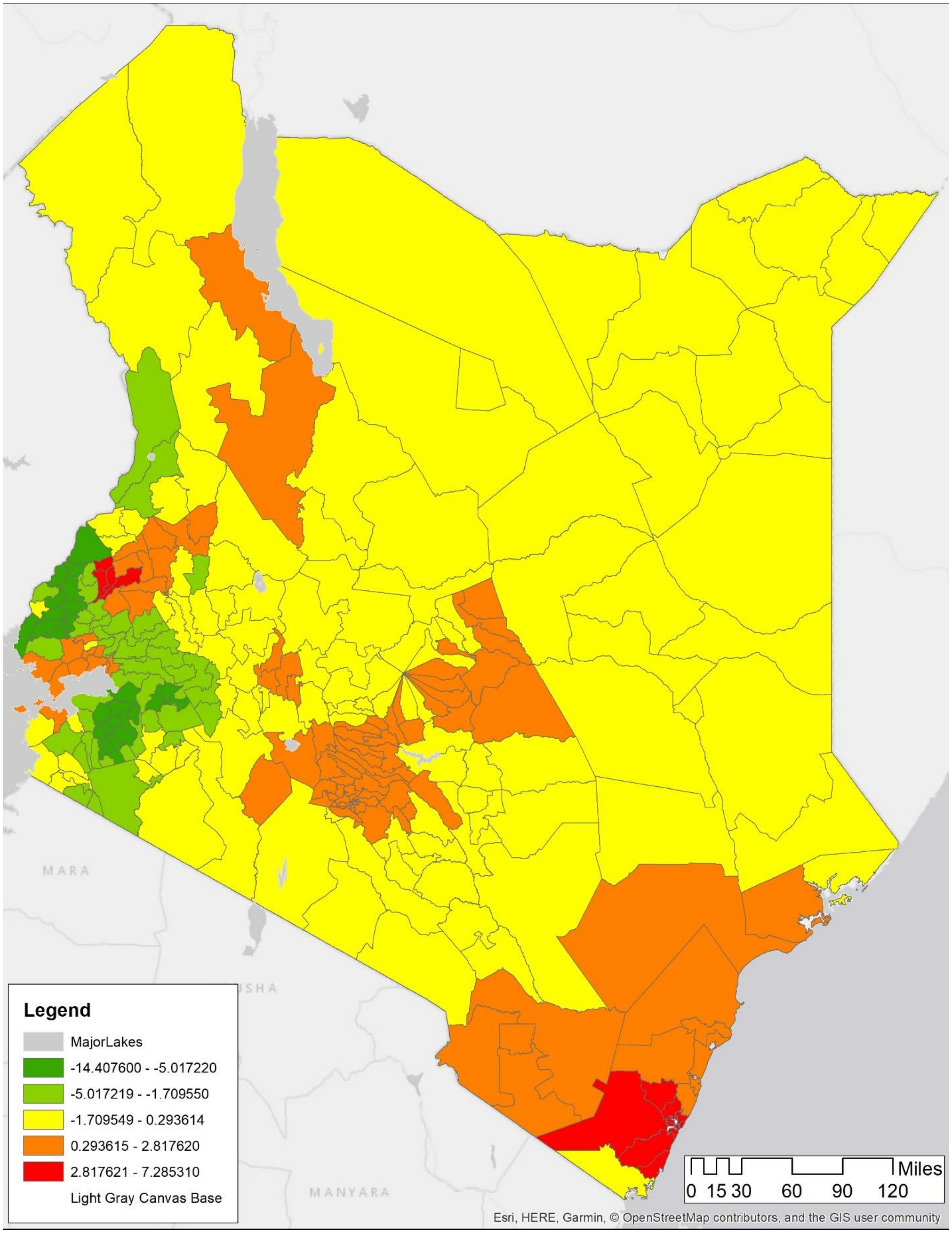

Figure S8. Map of GWR coefficients of proximity to water in 2005 (aggregated to county level)

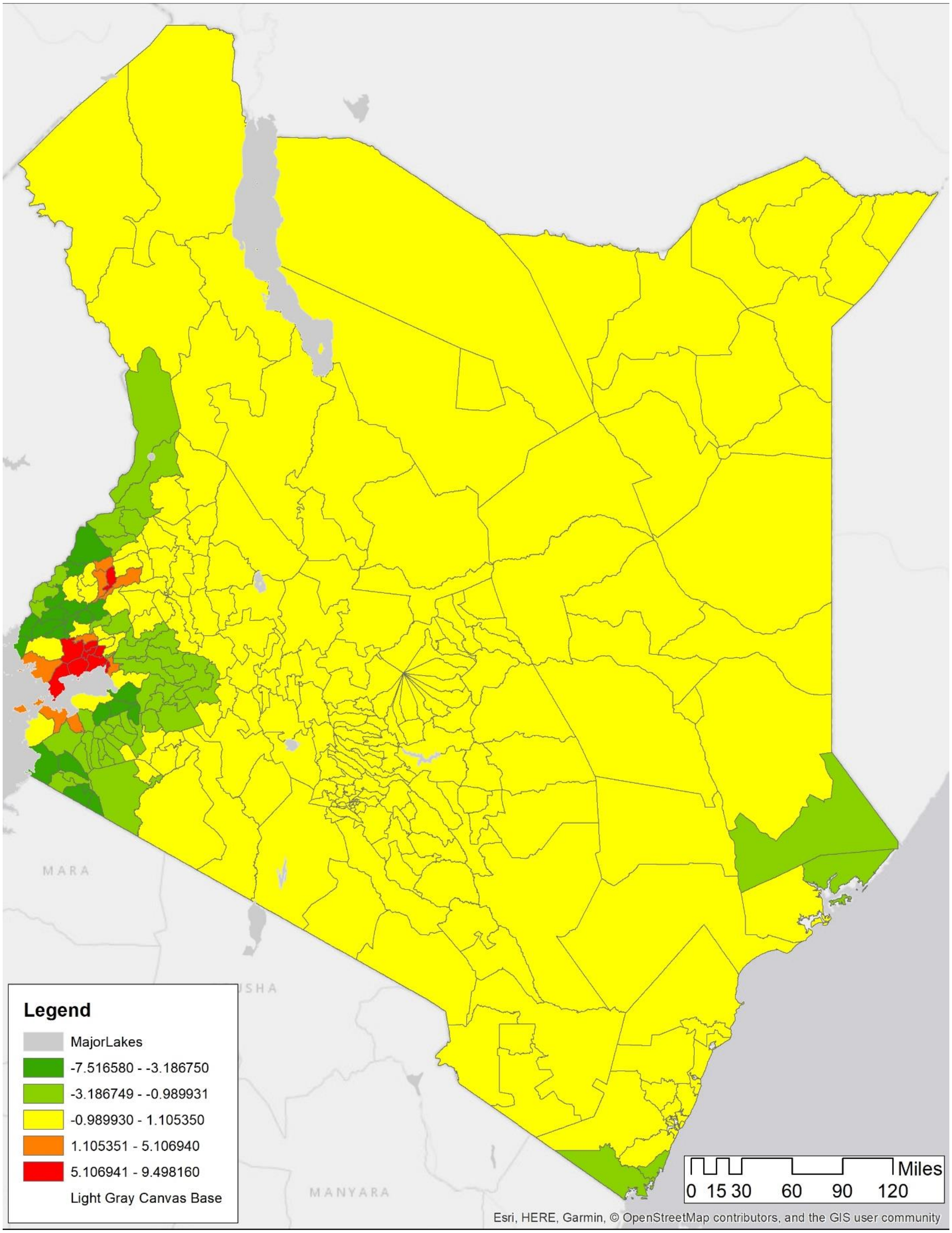

Figure S9. Map of GWR coefficients of proximity to water in 2010 (aggregated to county level)

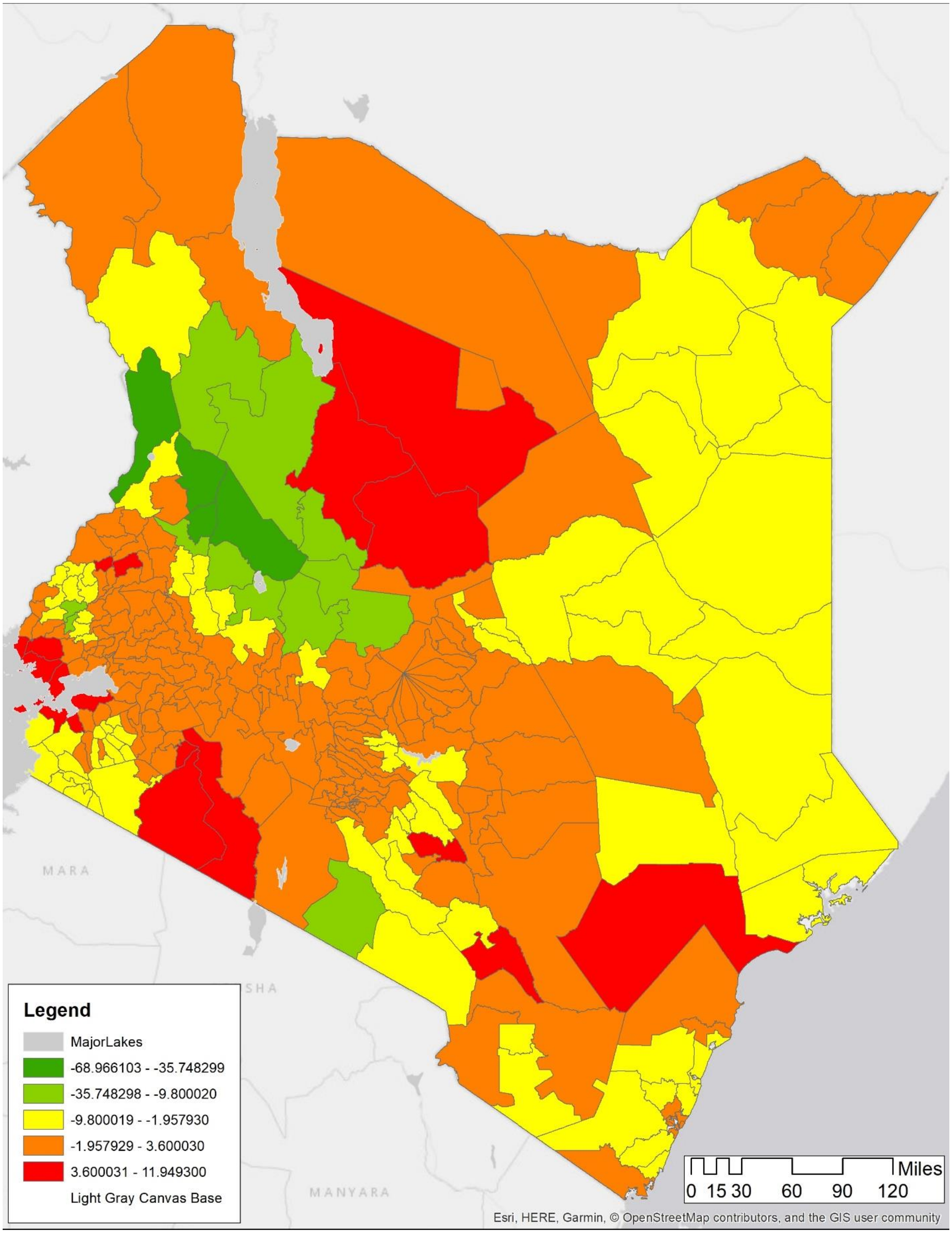

Figure 10. Map of GWR coefficients of population density in 2000 (aggregated to county level)

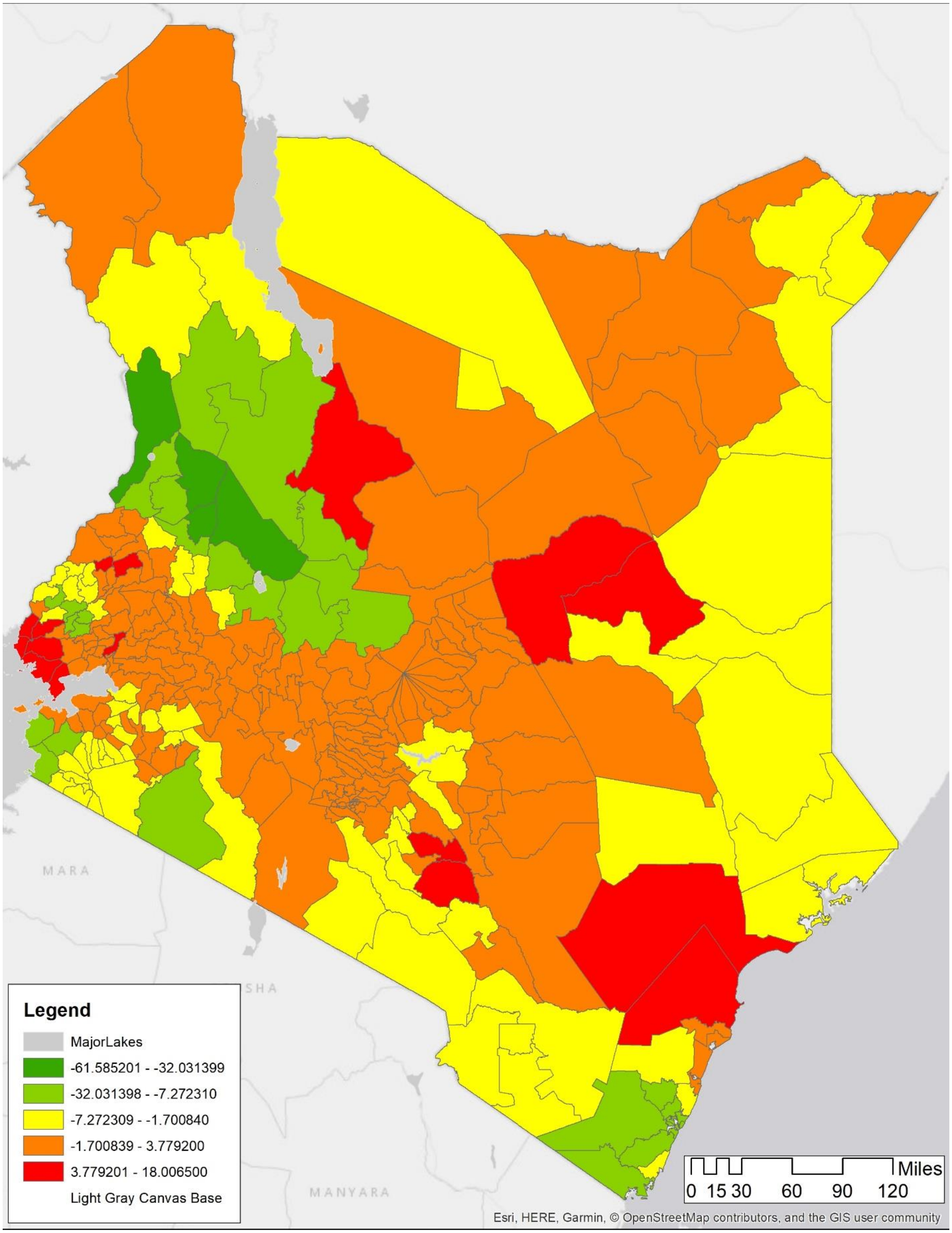

Figure S11. Map of GWR coefficients of population density in 2005 (aggregated to county level)

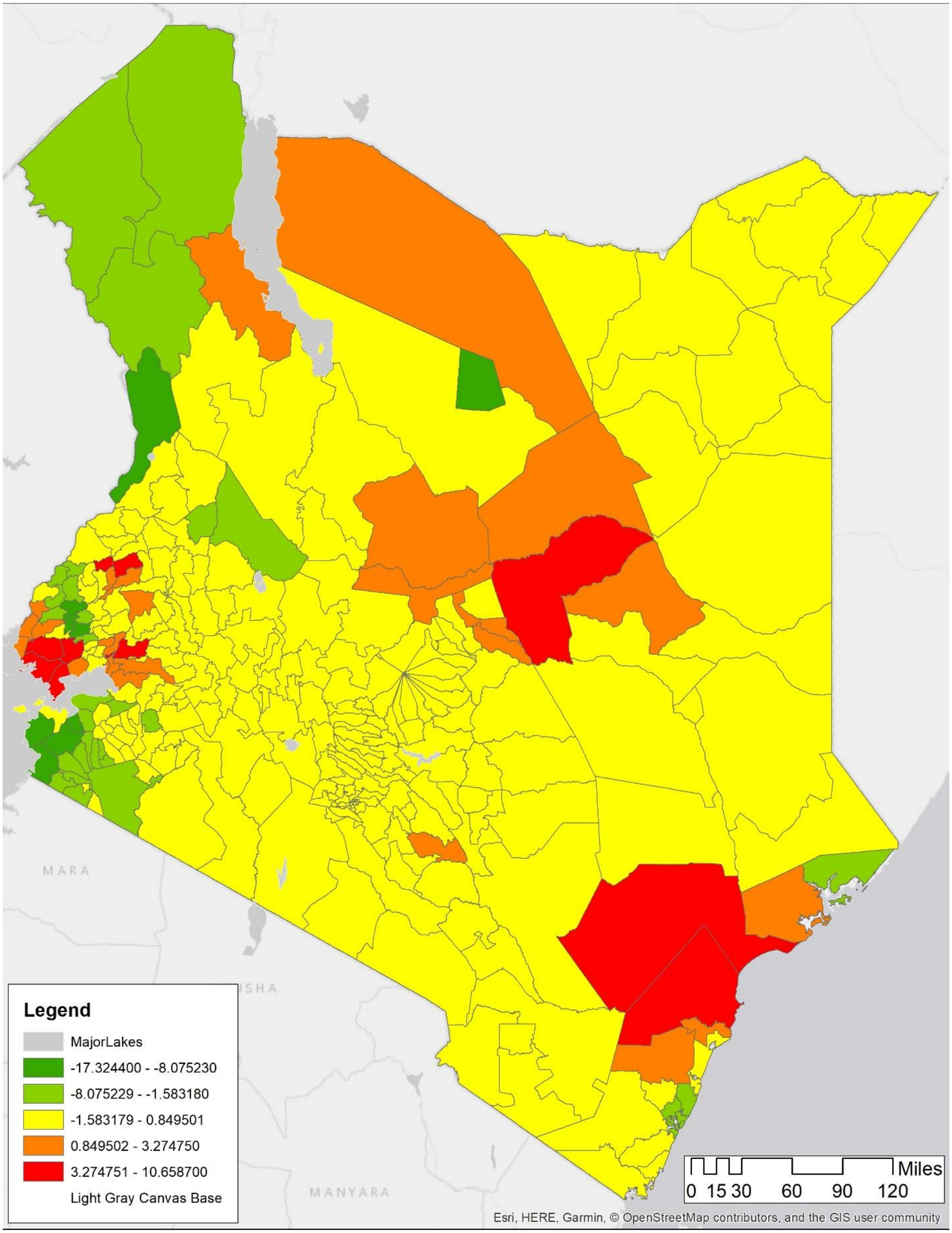

Figure S12. Map of GWR coefficients of population density in 2010 (aggregated to county level)

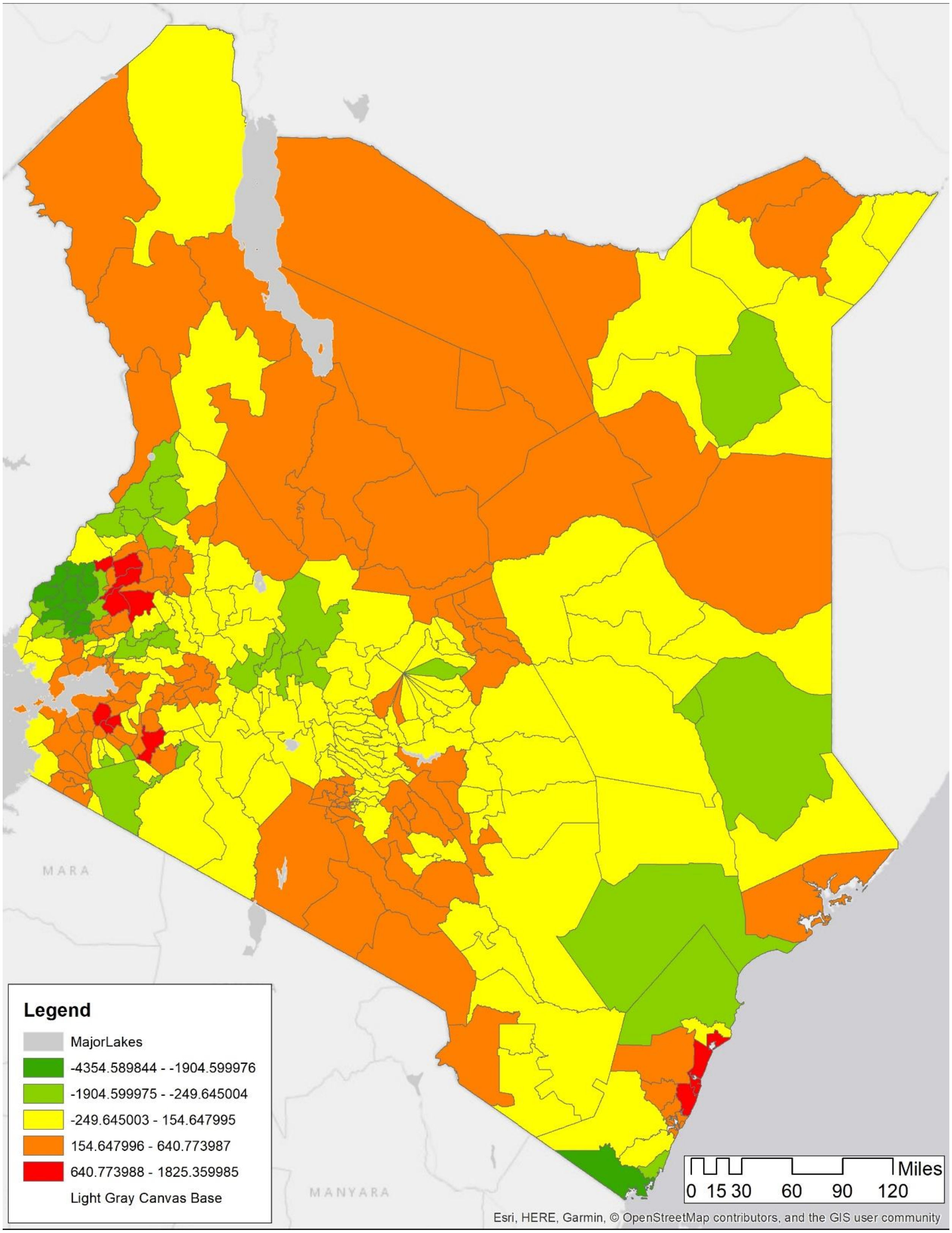

Figure S13. Map of GWR coefficients of EVI (enhanced vegetation index) in 2000 (aggregated to county level)

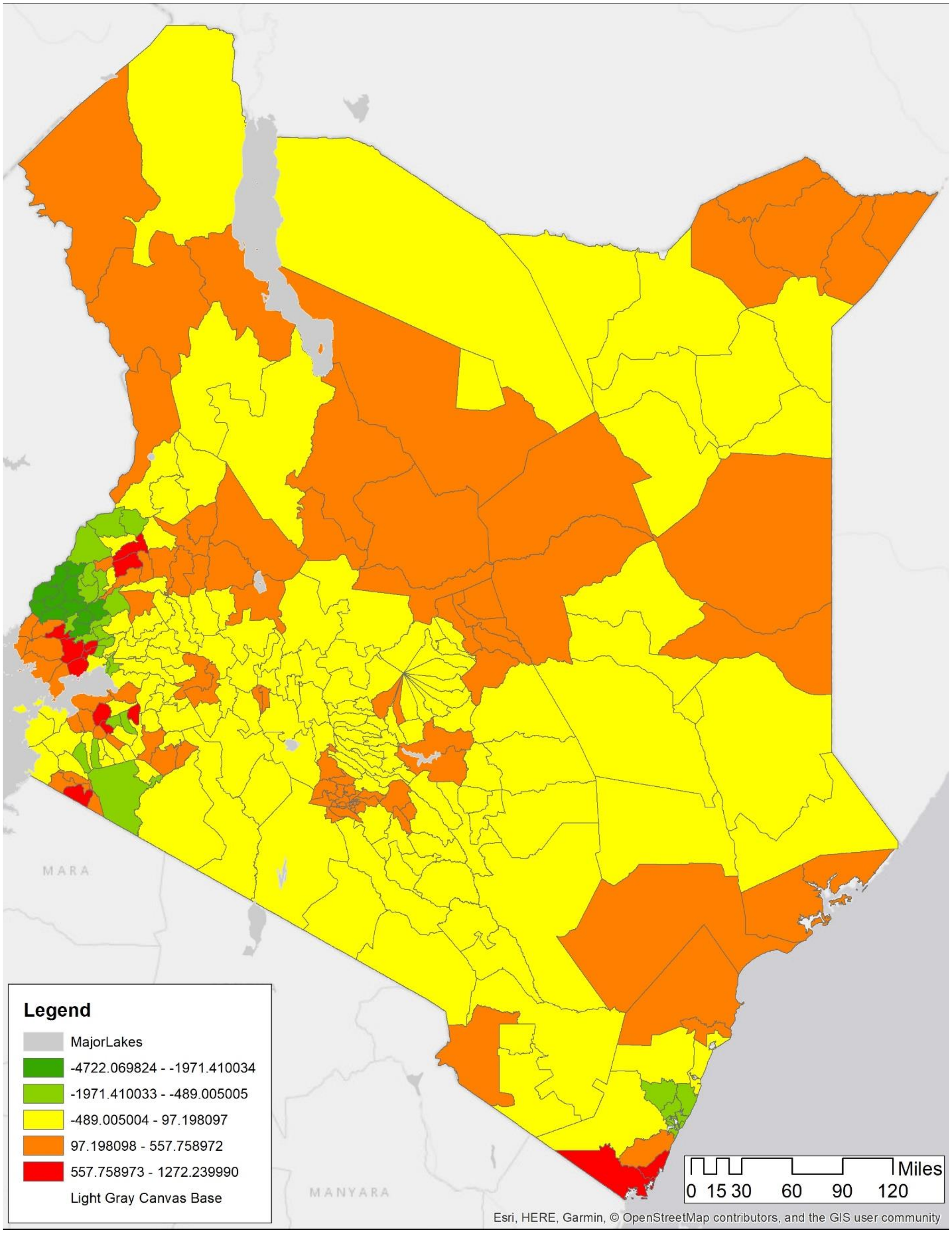

Figure S14. Map of GWR coefficients of EVI (enhanced vegetation index) in 2005 (aggregated to county level)

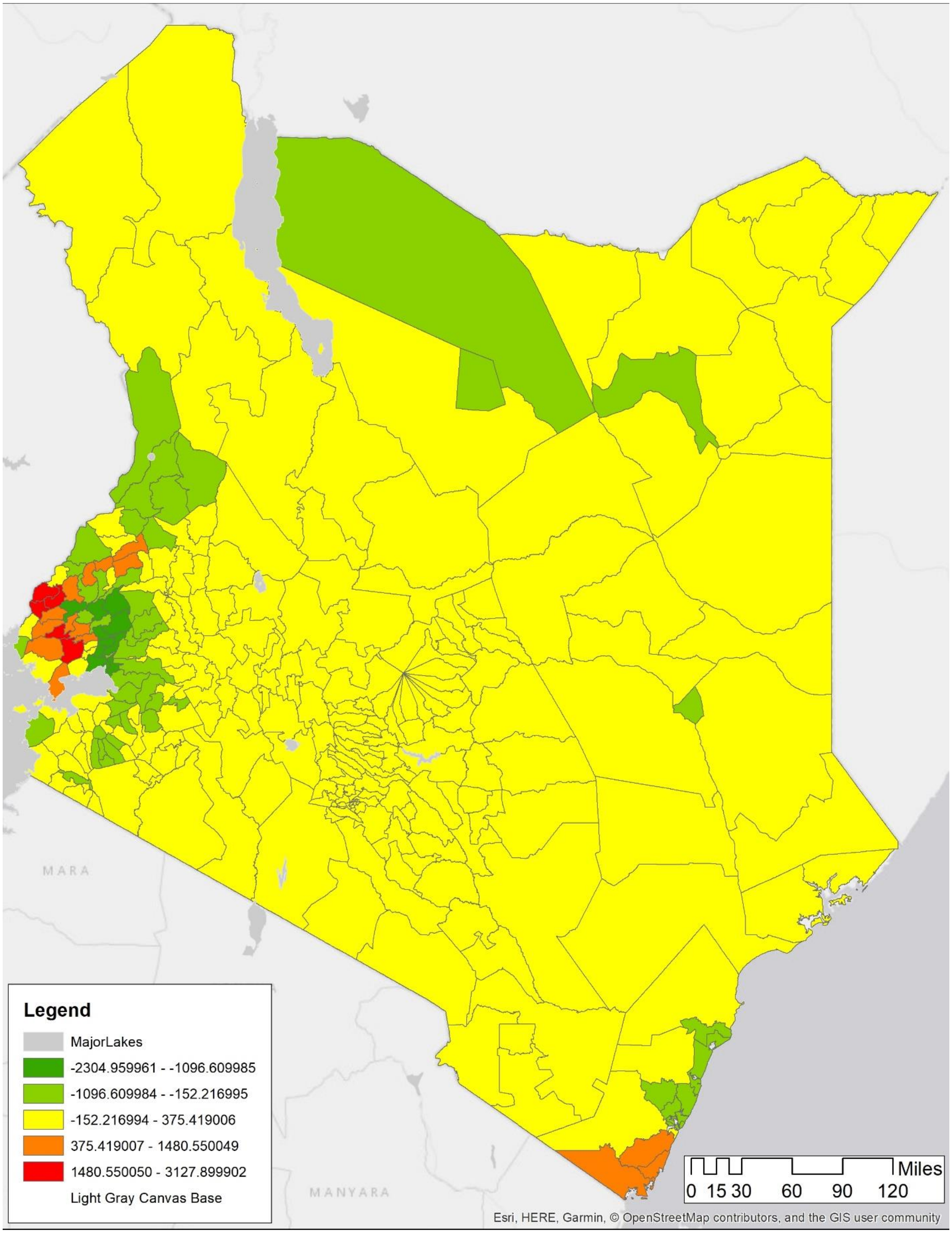

Figure S15. Map of GWR coefficients of EVI (enhanced vegetation index) in 2010 (aggregated to county level)

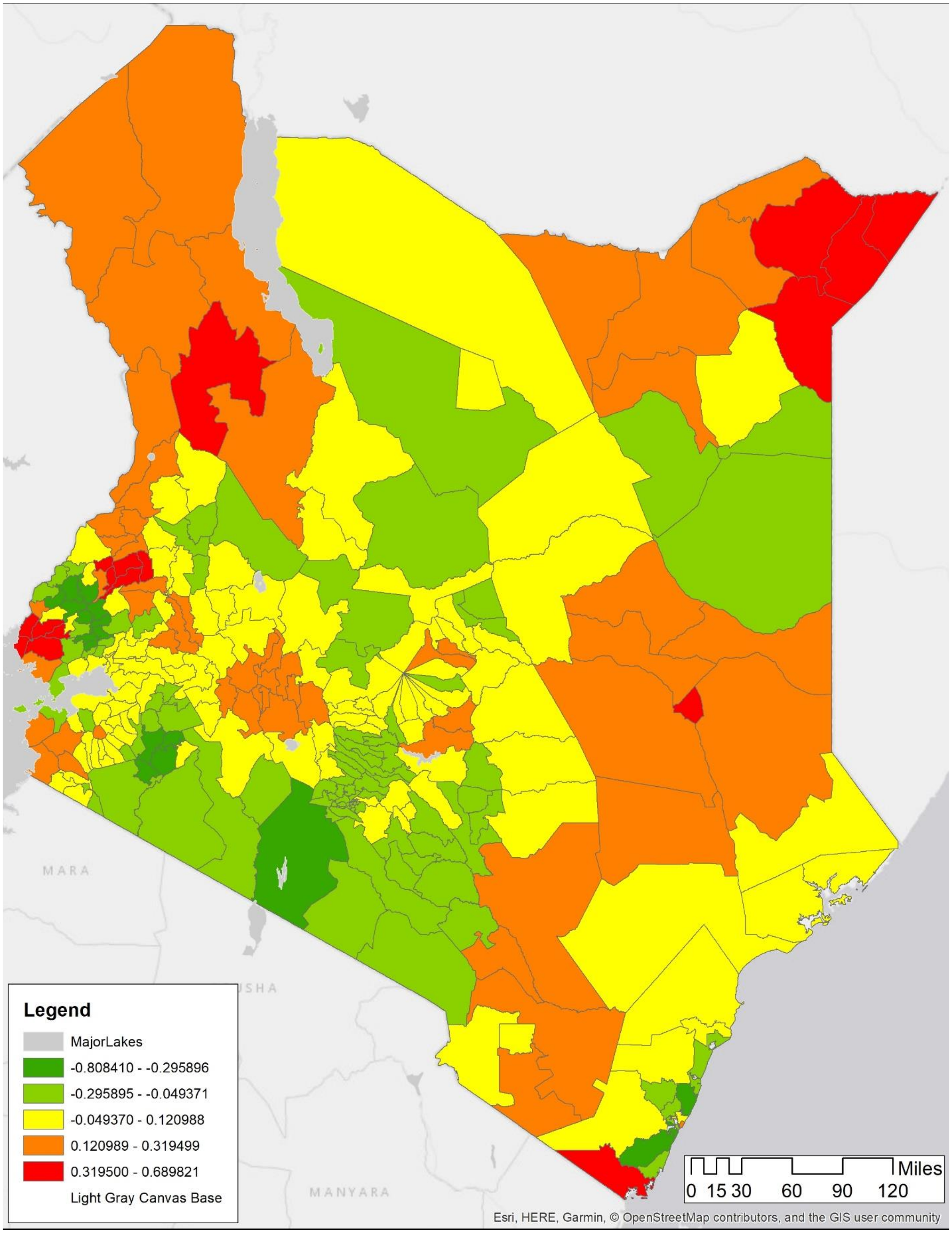

Figure S16. Map of GWR coefficients of rainfall in 2000 (aggregated to county level)

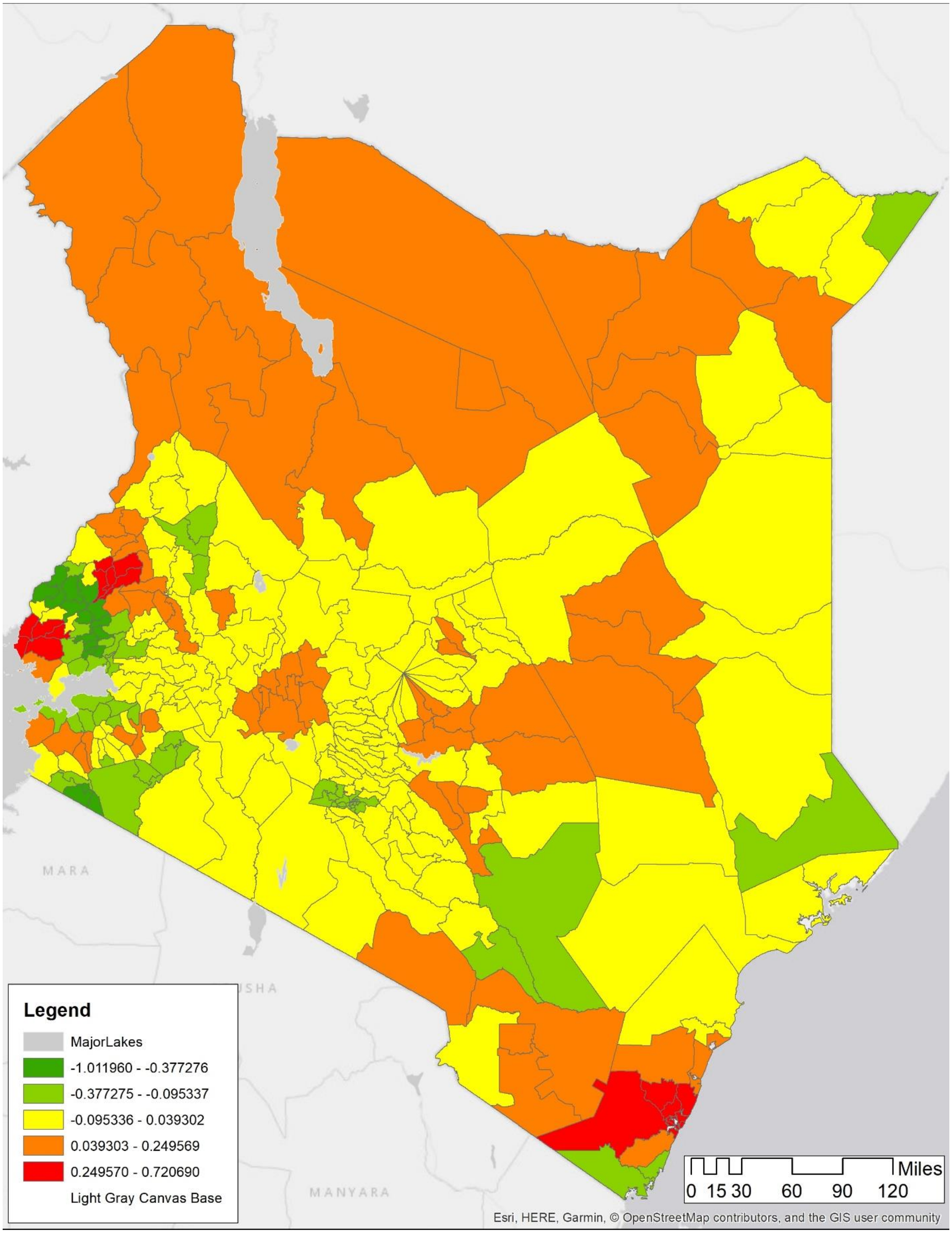

Figure S17. Map of GWR coefficients of rainfall in 2005 (aggregated to county level)

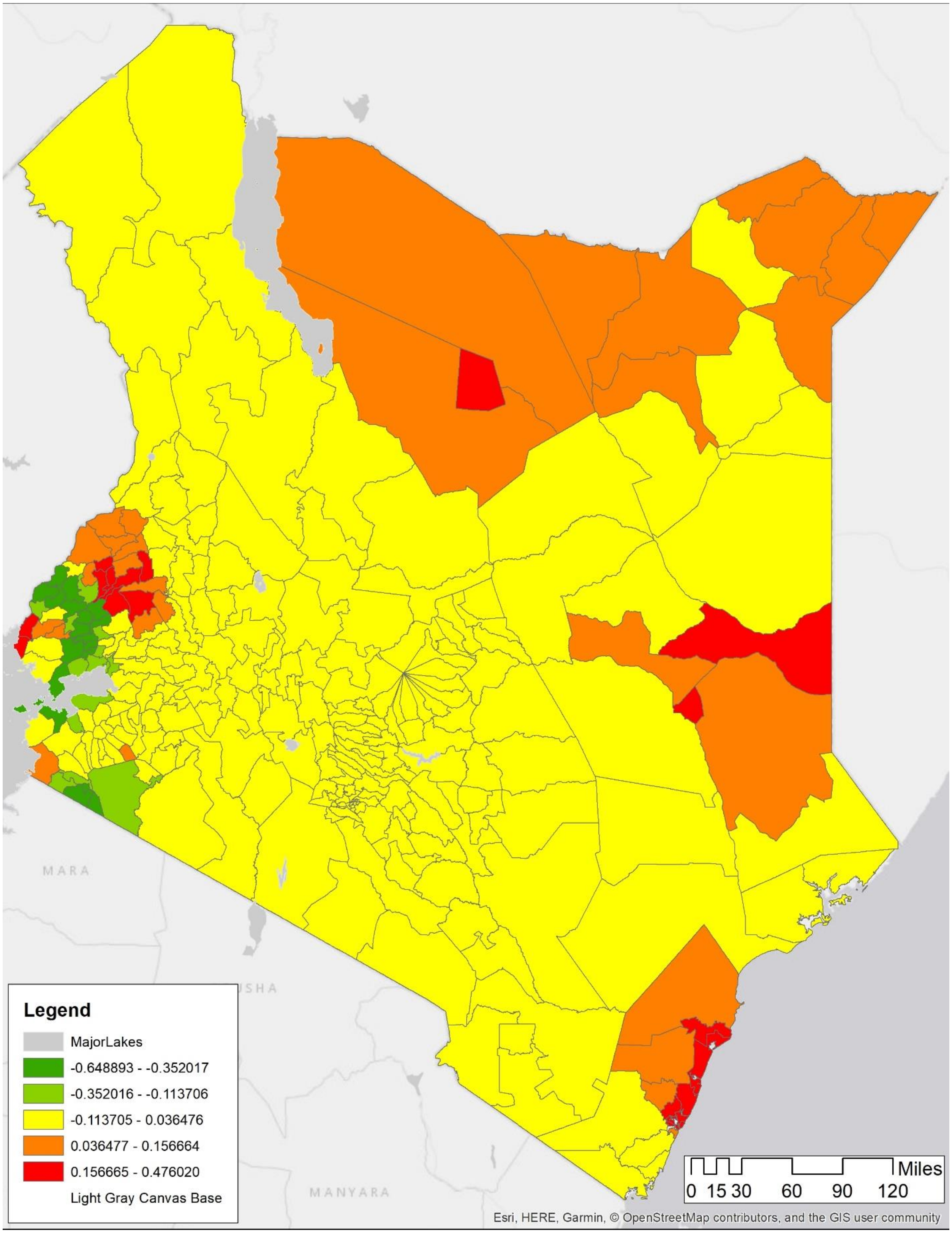

Figure S18. Map of GWR coefficients of rainfall in 2010 (aggregated to county level)

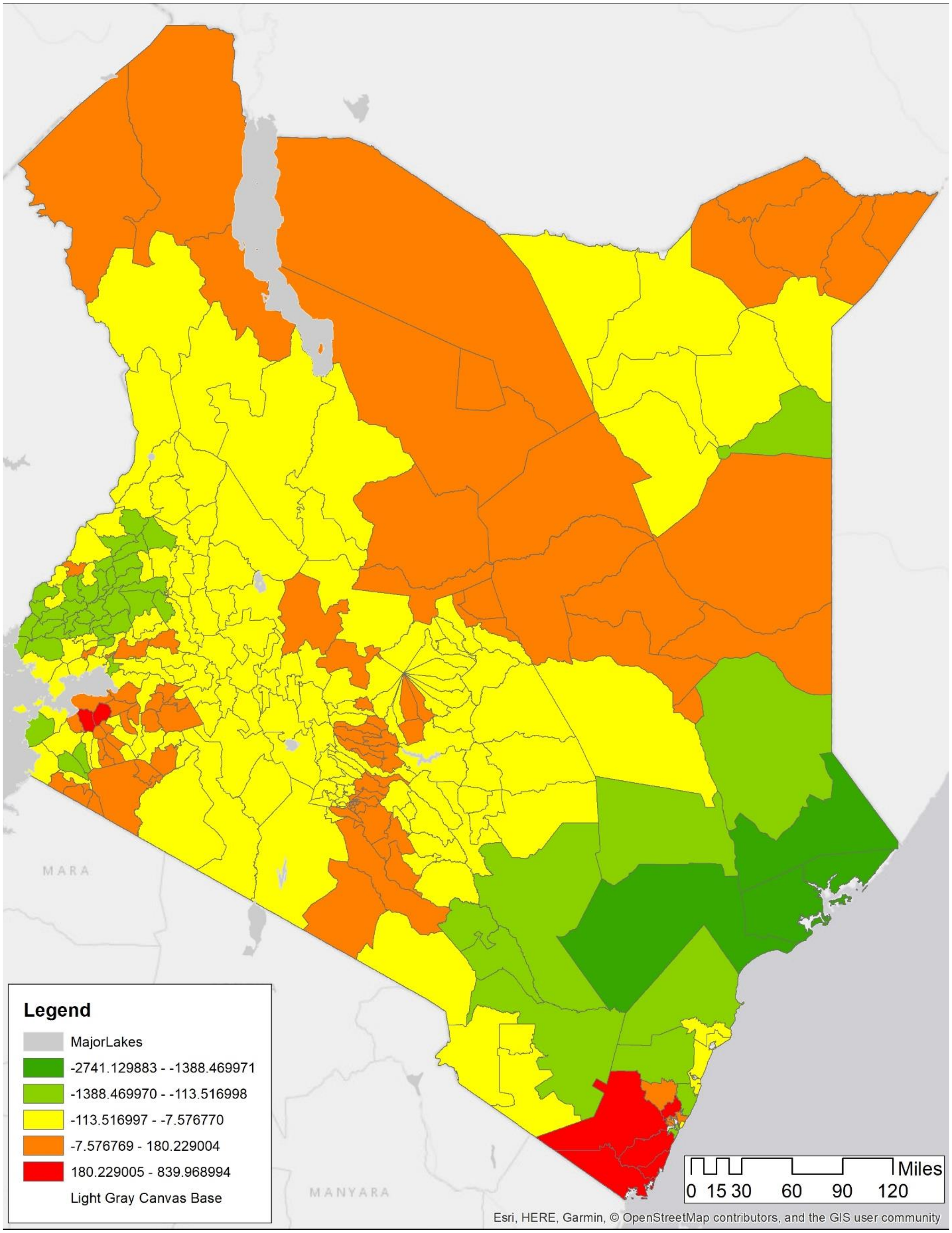

Figure S19. Map of GWR coefficients of elevation in 2000 (aggregated to county level)

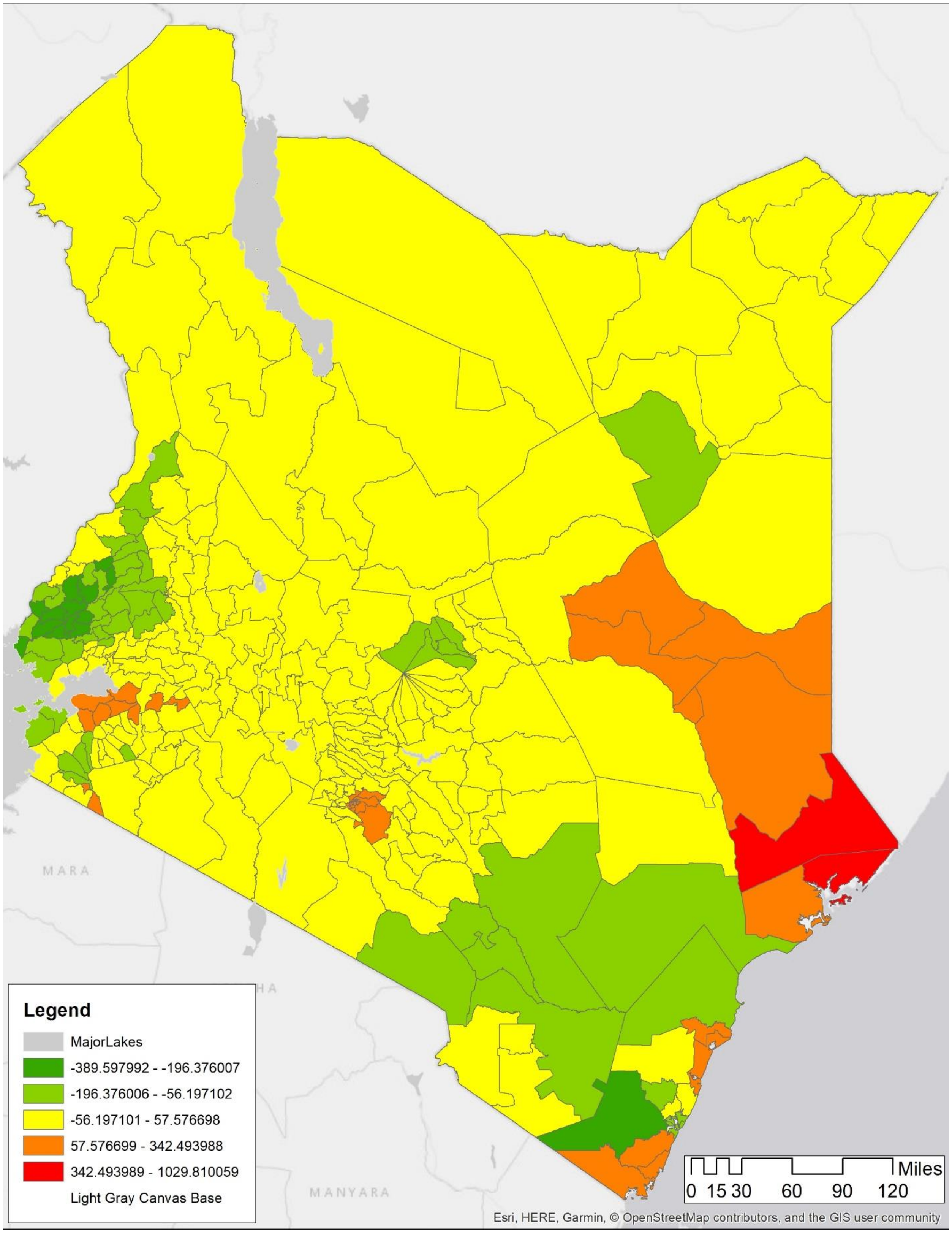

Figure S20. Map of GWR coefficients of elevation in 2005 (aggregated to county level)

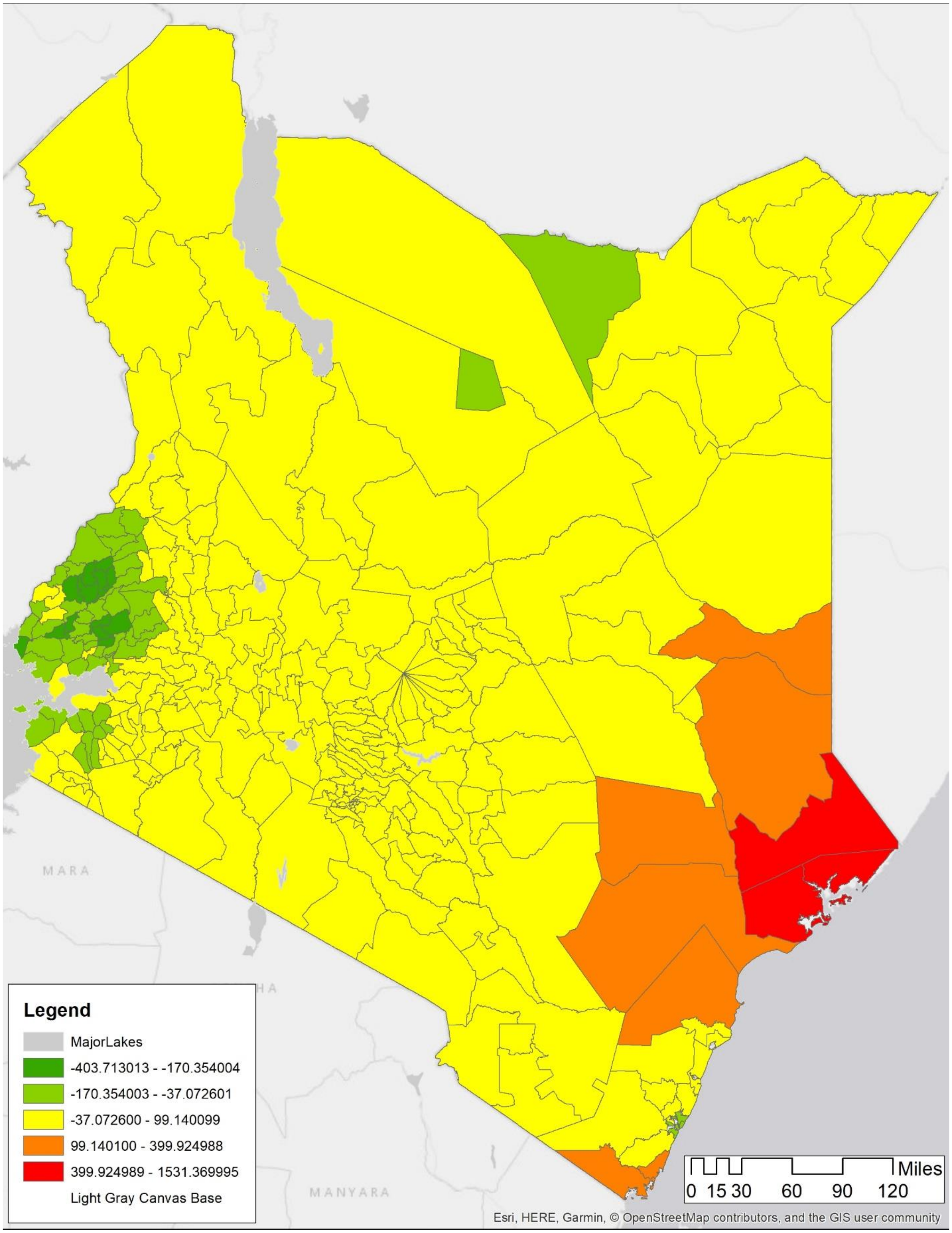

Figure S21. Map of GWR coefficients of elevation in 2010 (aggregated to county level)

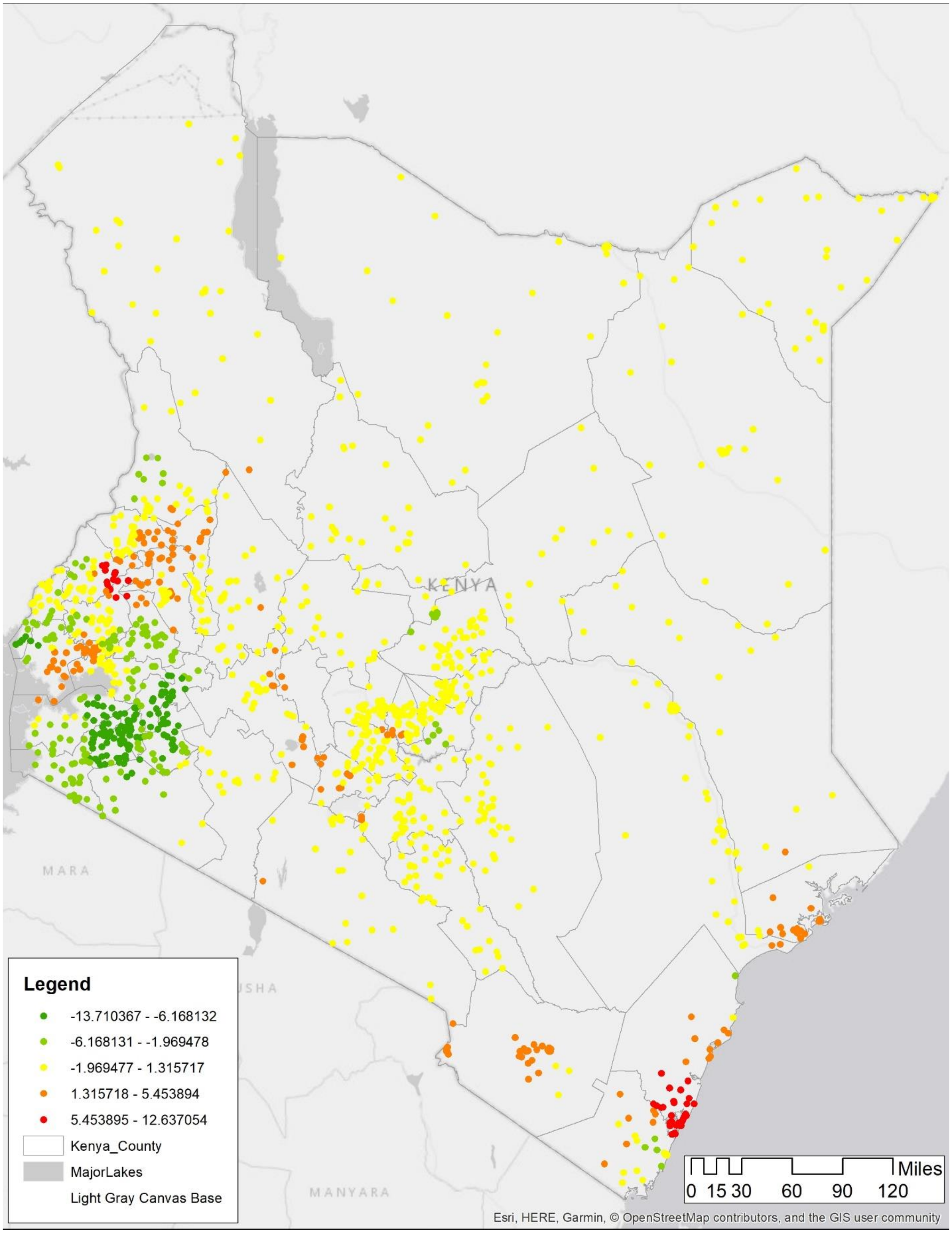

Figure S22. Map of GWR coefficients of proximity to water in 2000 (at DHS cluster)

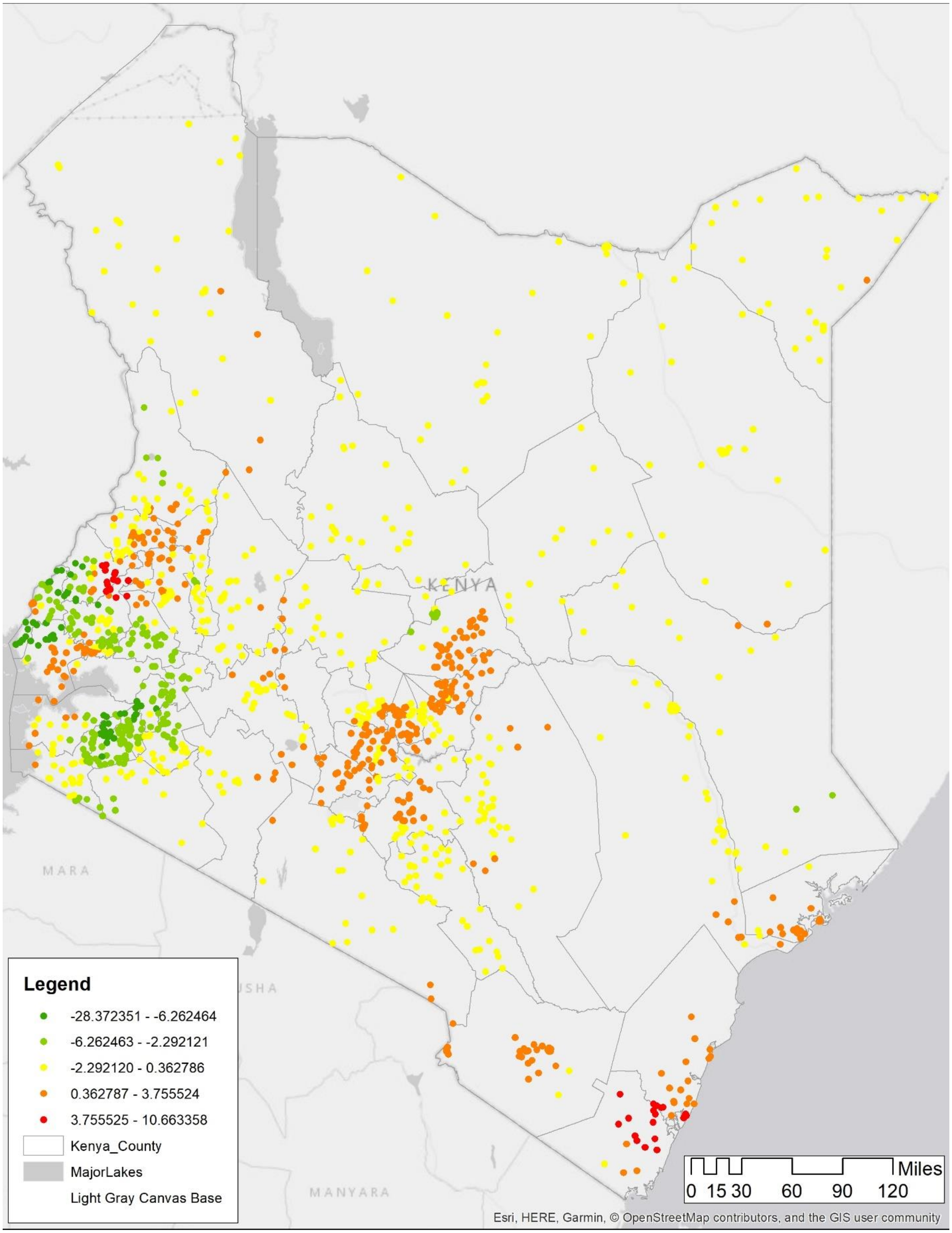

Figure S23. Map of GWR coefficients of proximity to water in 2005 (at DHS cluster)

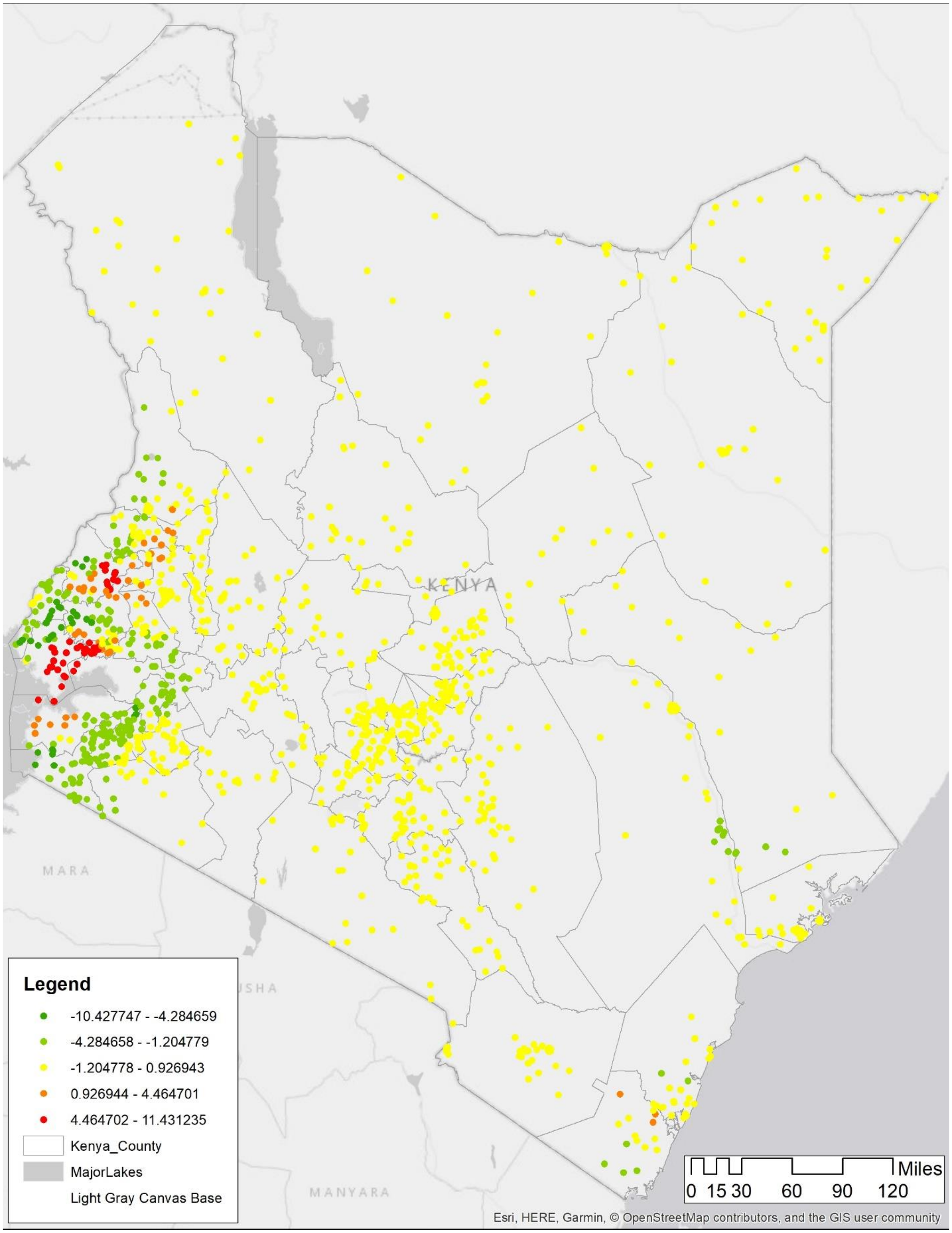

Figure S24. Map of GWR coefficients of proximity to water in 2010 (at DHS cluster)

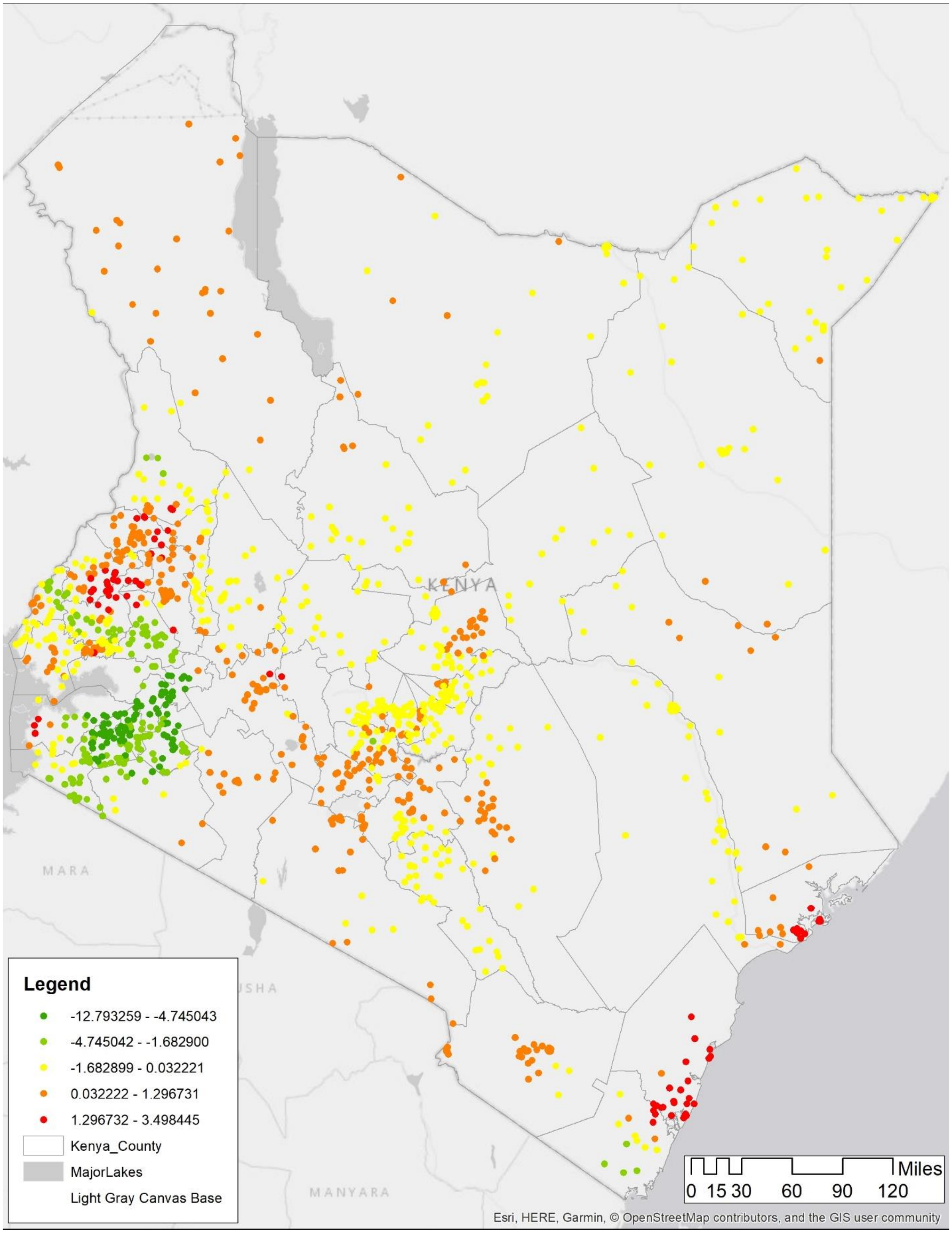

Figure S25. Map of GWR coefficients of proximity to water in 2015 (at DHS cluster)

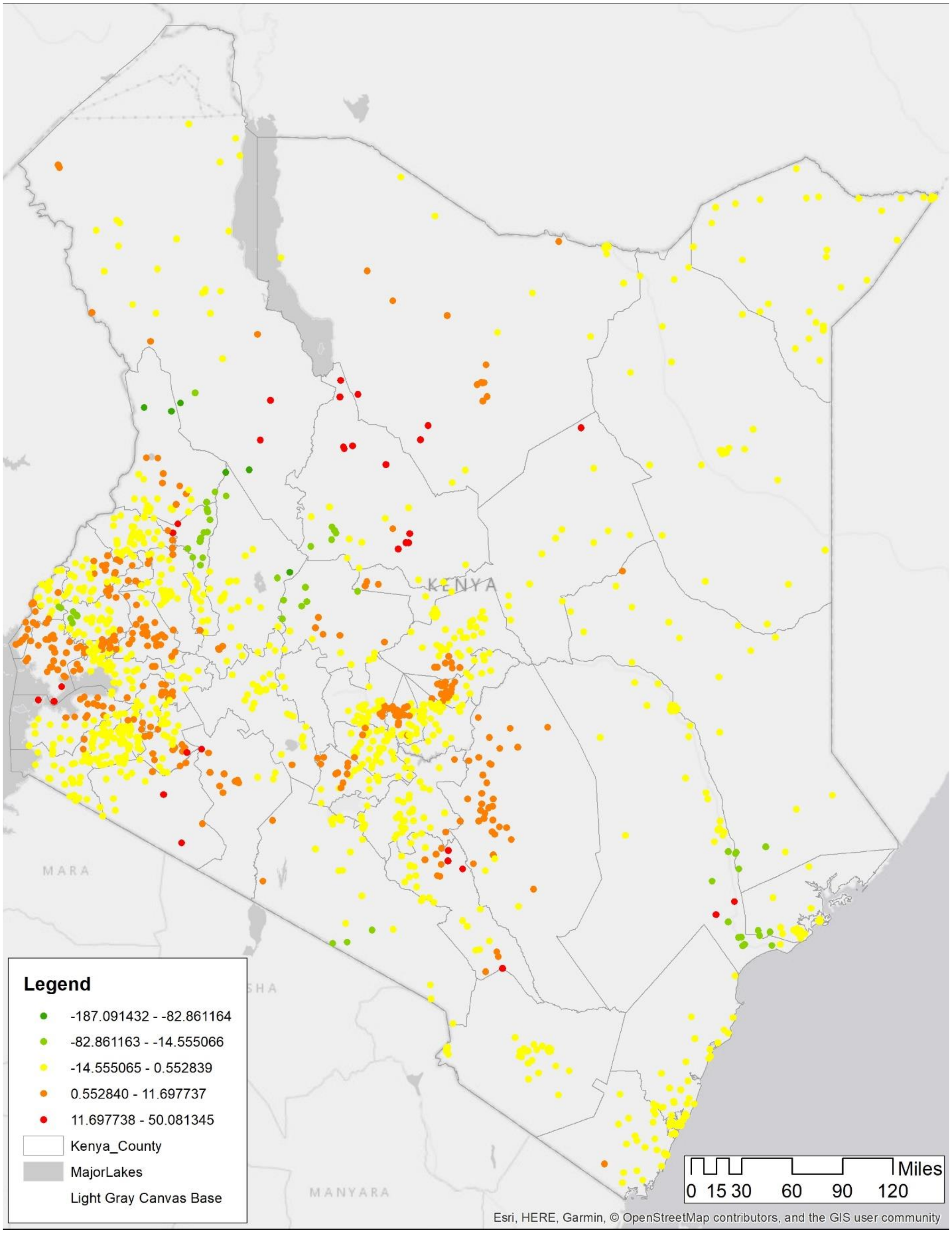

Figure S26. Map of GWR coefficients of population density in 2000 (at DHS cluster)

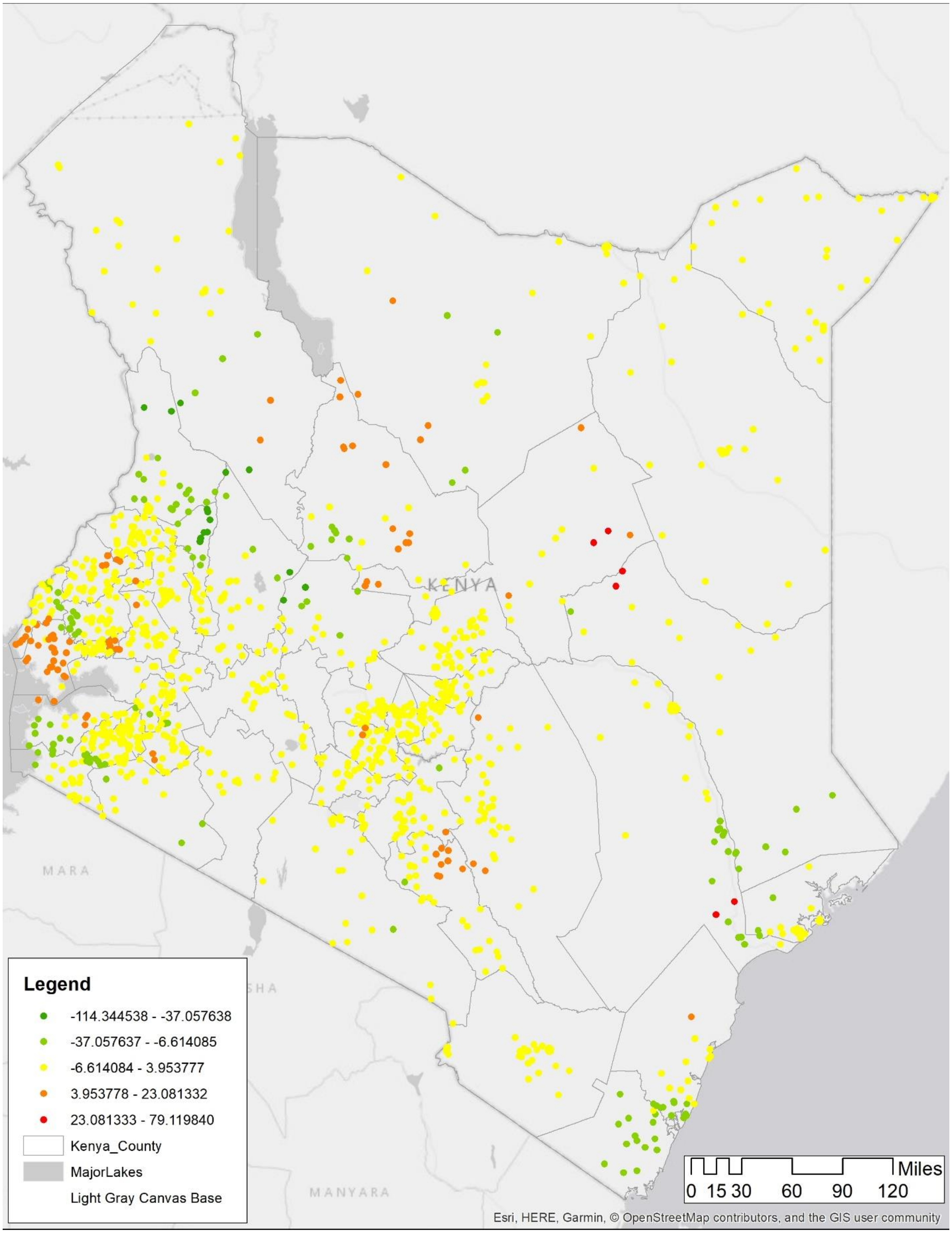

Figure S27. Map of GWR coefficients of population density in 2005 (at DHS cluster)

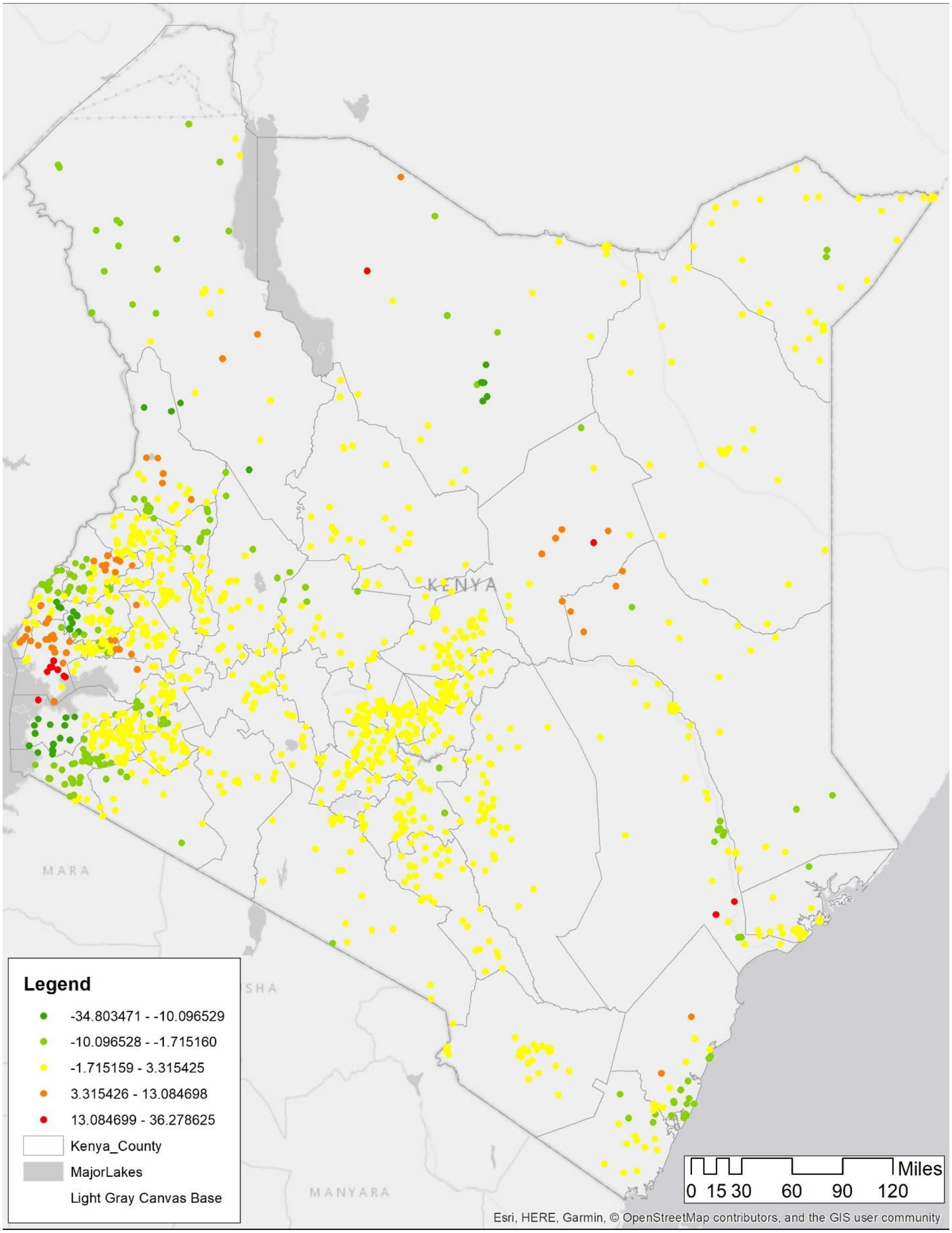

Figure S28. Map of GWR coefficients of population density in 2010 (at DHS cluster)

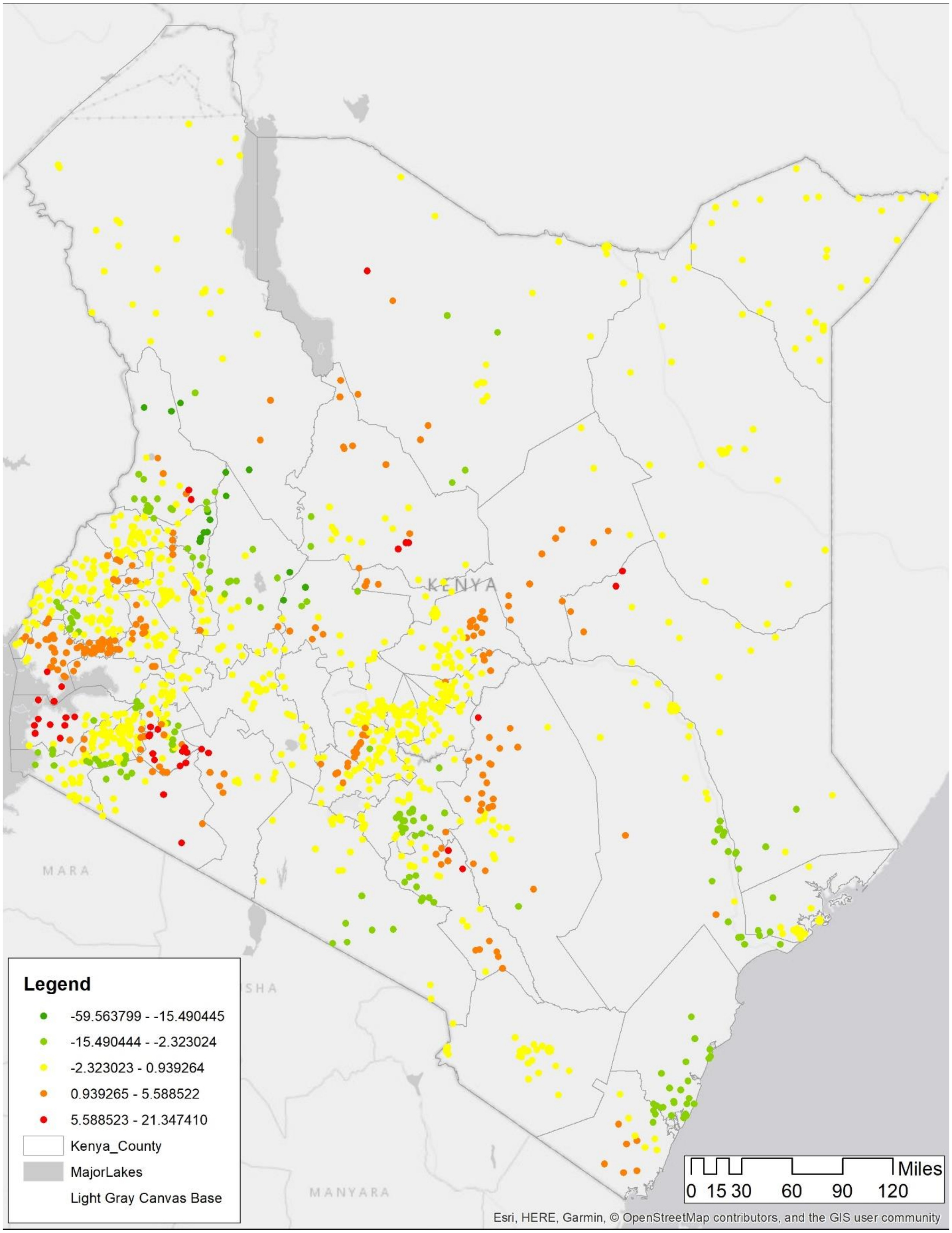

Figure S29. Map of GWR coefficients of population density in 2015 (at DHS cluster)

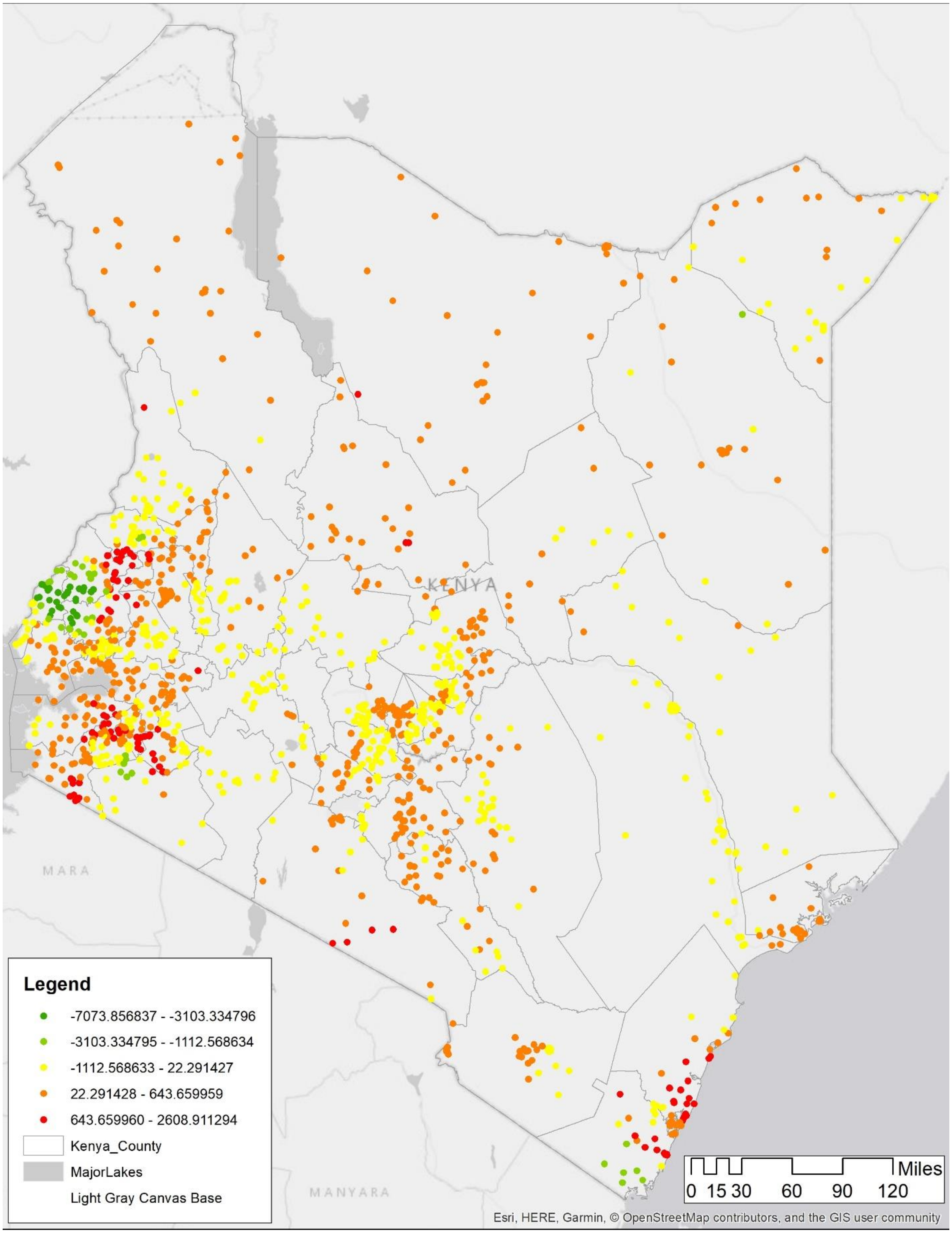

Figure S30. Map of GWR coefficients of EVI (enhanced vegetation index) in 2000 (at DHS cluster)

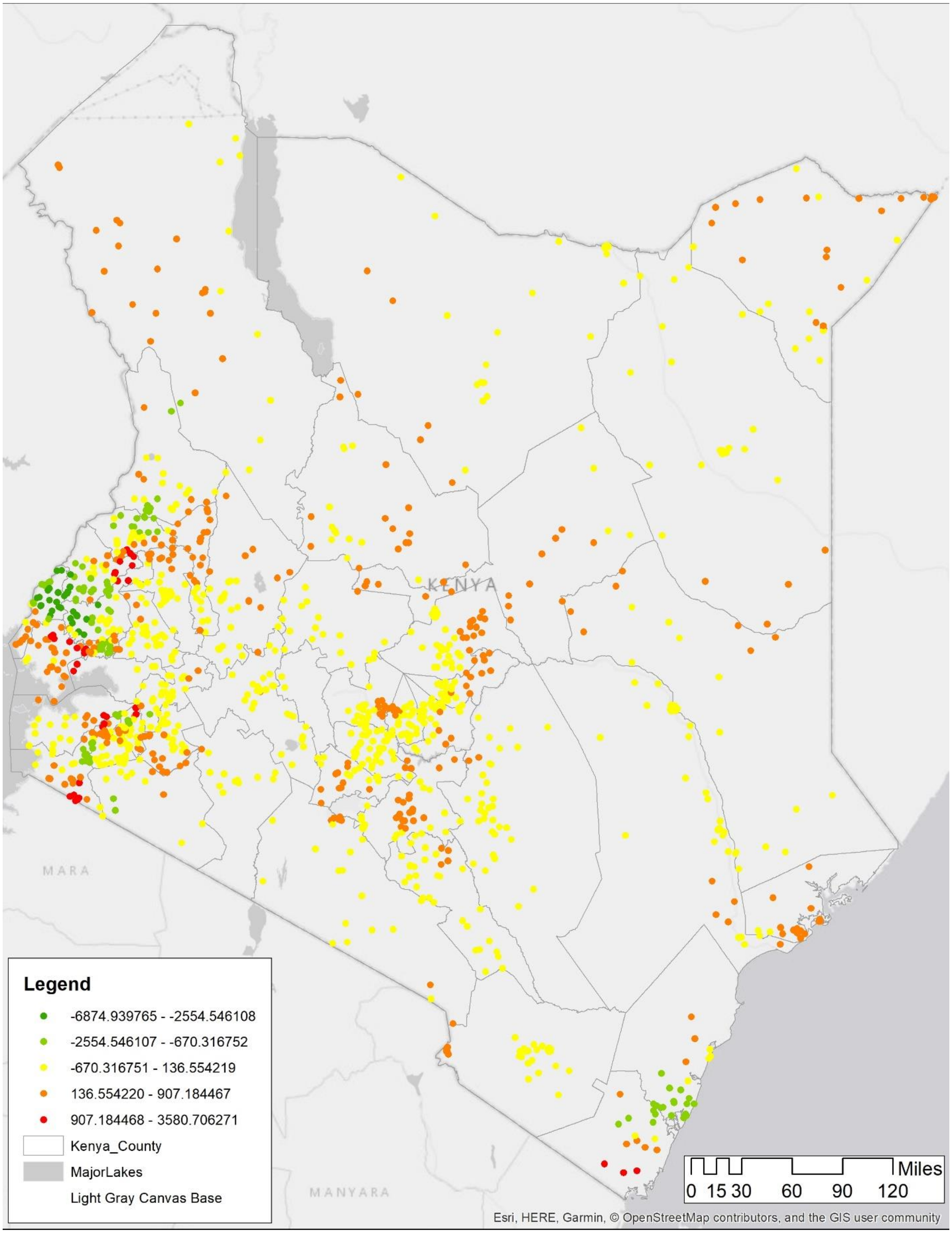

Figure S31. Map of GWR coefficients of EVI (enhanced vegetation index) in 2005 (at DHS cluster)

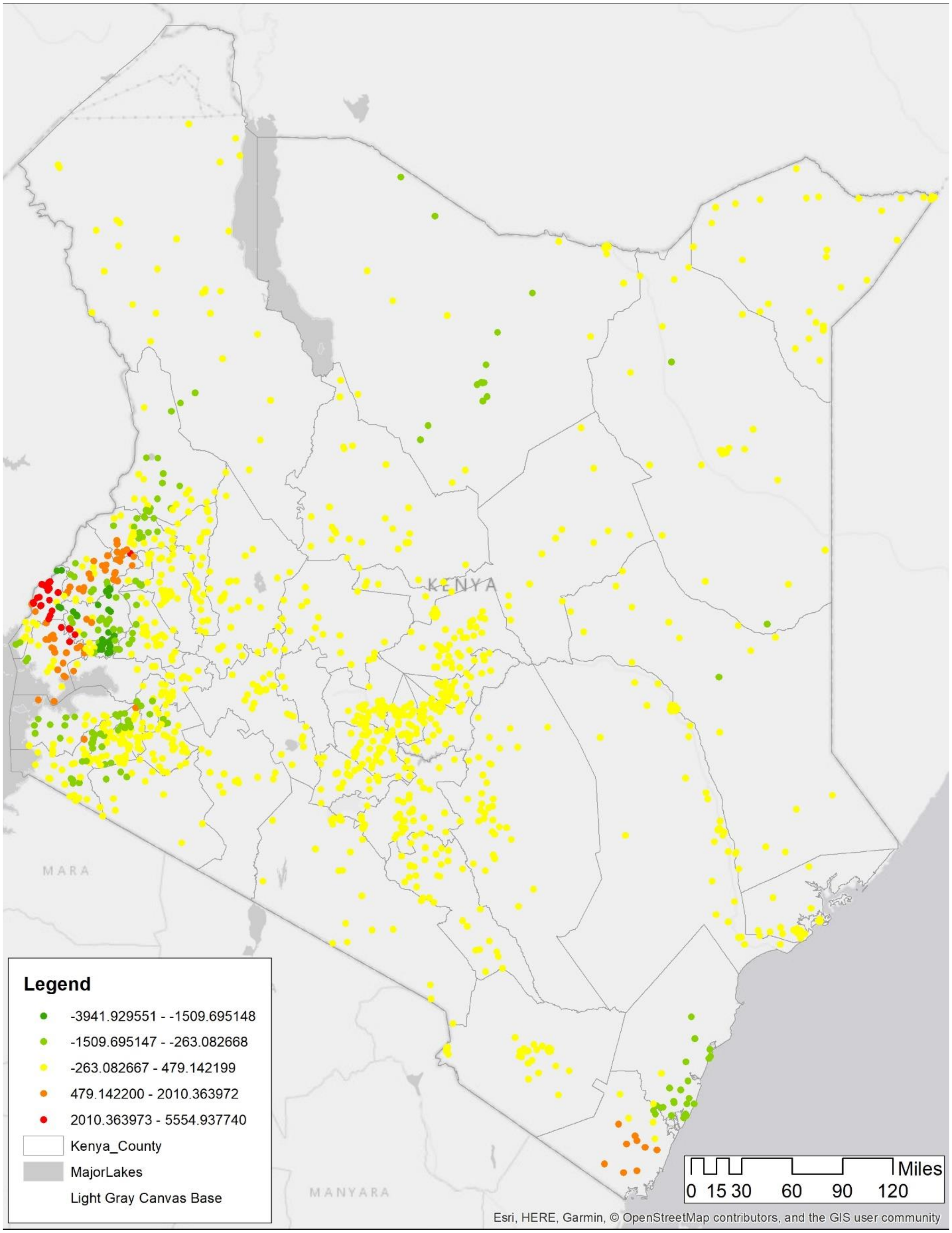

Figure S32. Map of GWR coefficients of EVI (enhanced vegetation index) in 2010 (at DHS cluster)

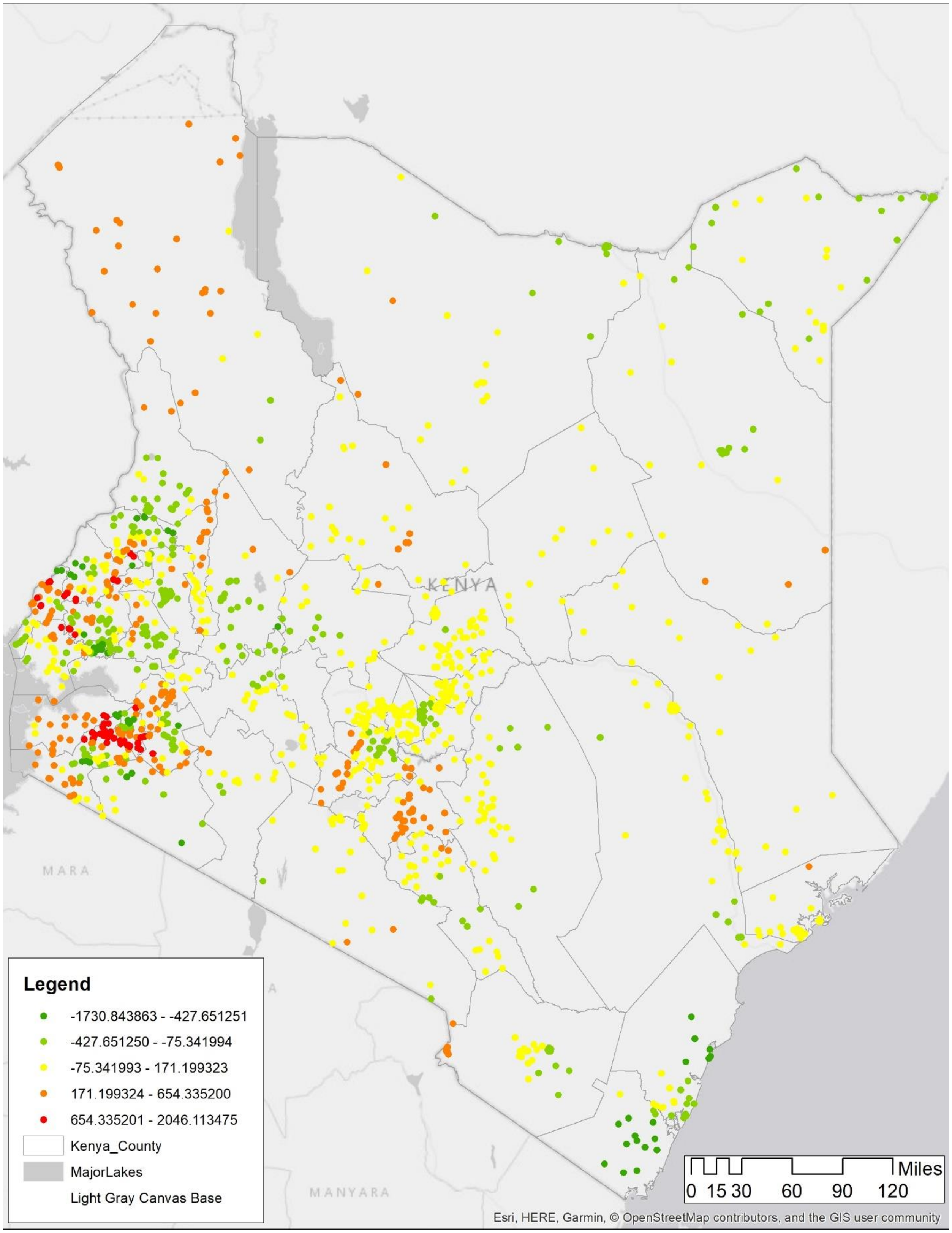

Figure S33. Map of GWR coefficients of EVI (enhanced vegetation index) in 2015 (at DHS cluster)

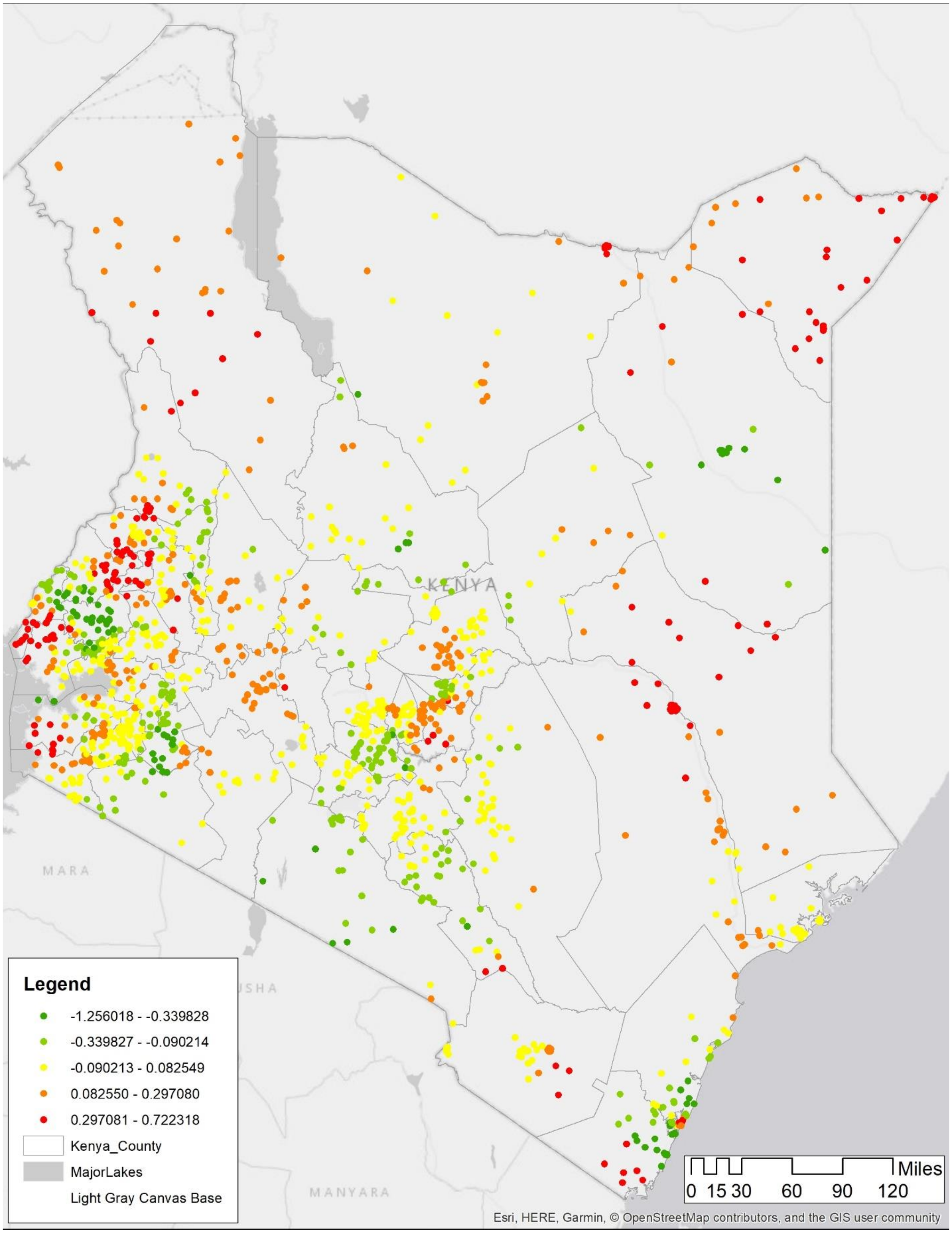

Figure S34. Map of GWR coefficients of rainfall in 2000 (at DHS cluster)

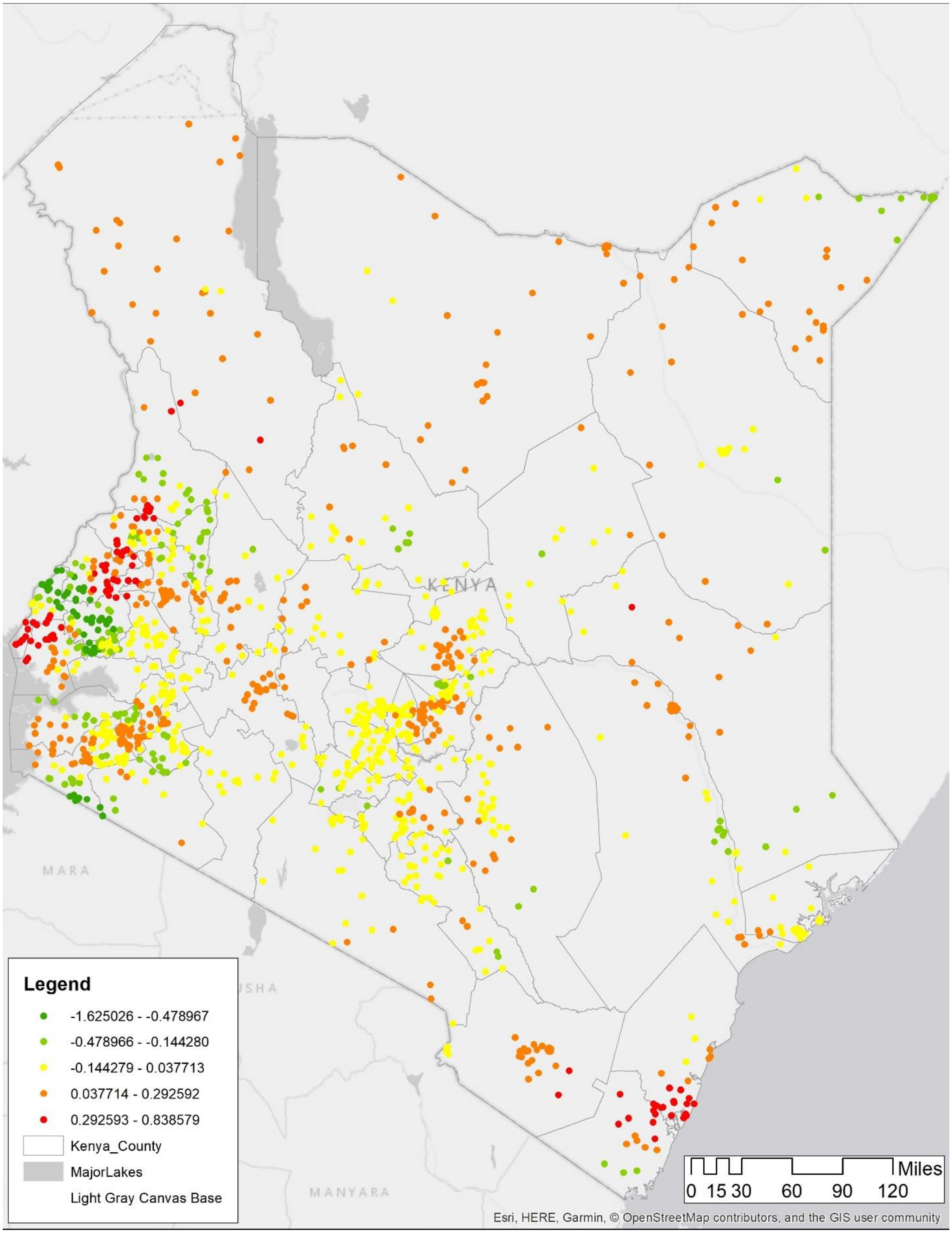

Figure S35. Map of GWR coefficients of rainfall in 2005 (at DHS cluster)

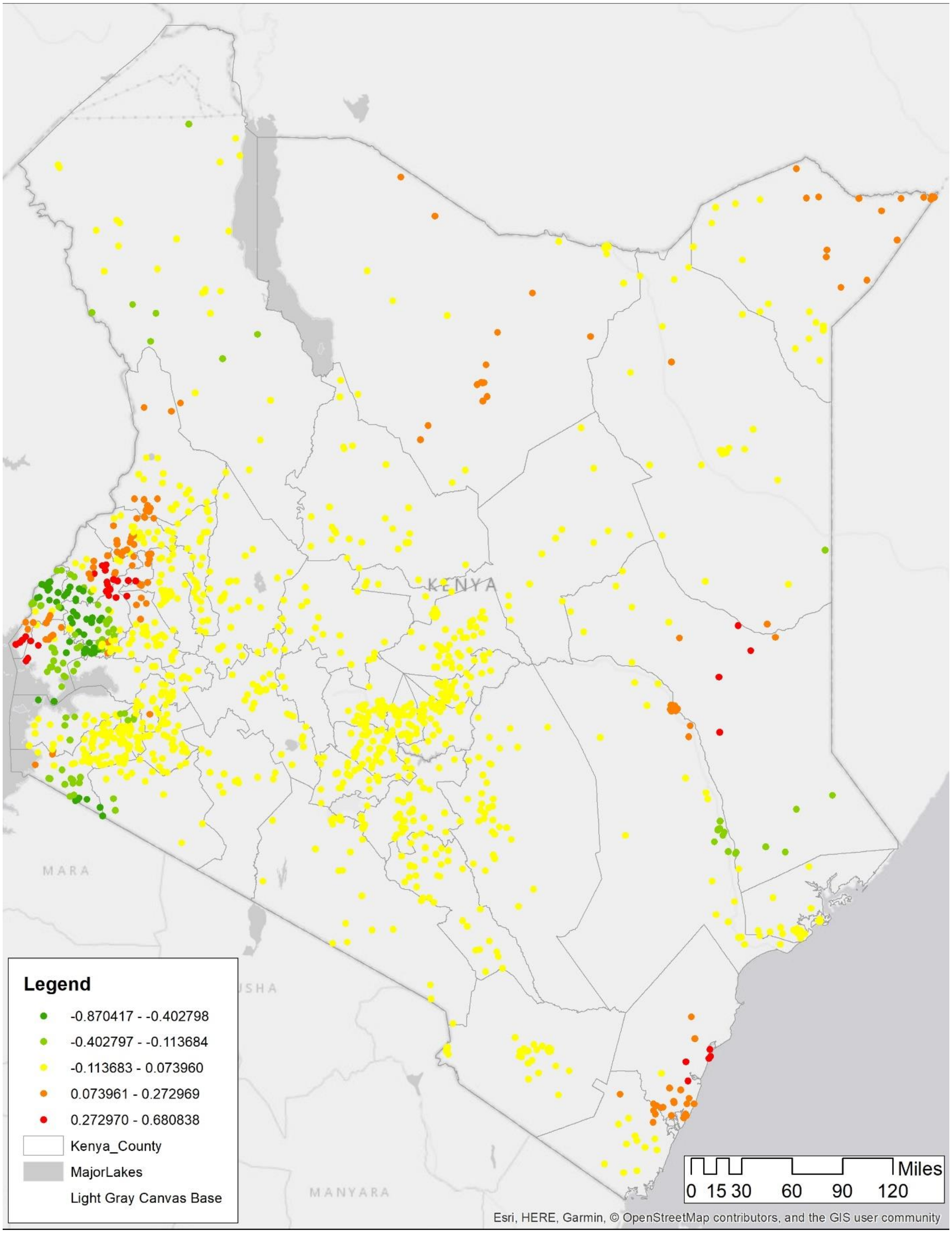

Figure S36. Map of GWR coefficients of rainfall in 2010 (at DHS cluster)

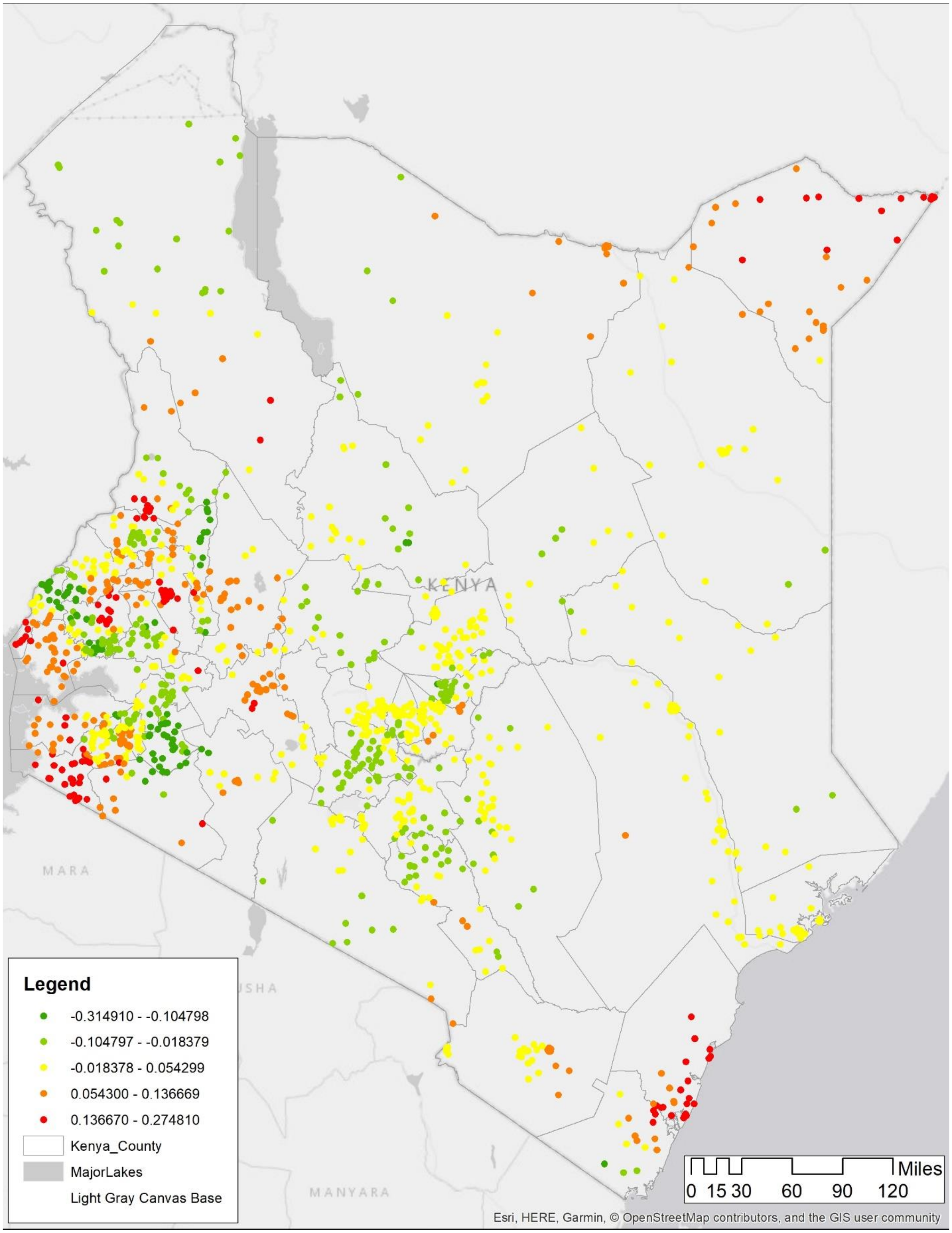

Figure S37. Map of GWR coefficients of rainfall in 2015 (at DHS cluster)

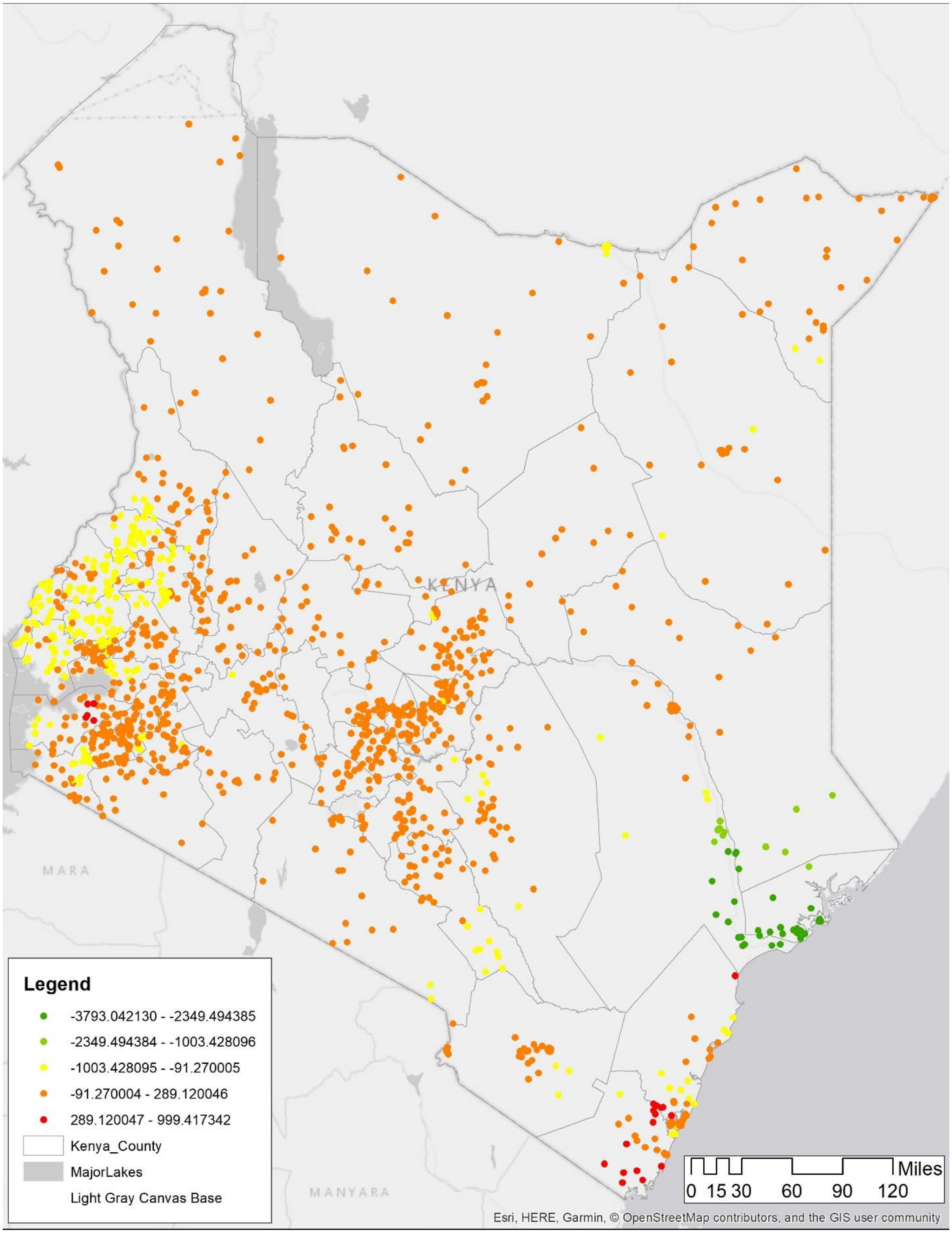

Figure S38. Map of GWR coefficients of elevation in 2000 (at DHS cluster)

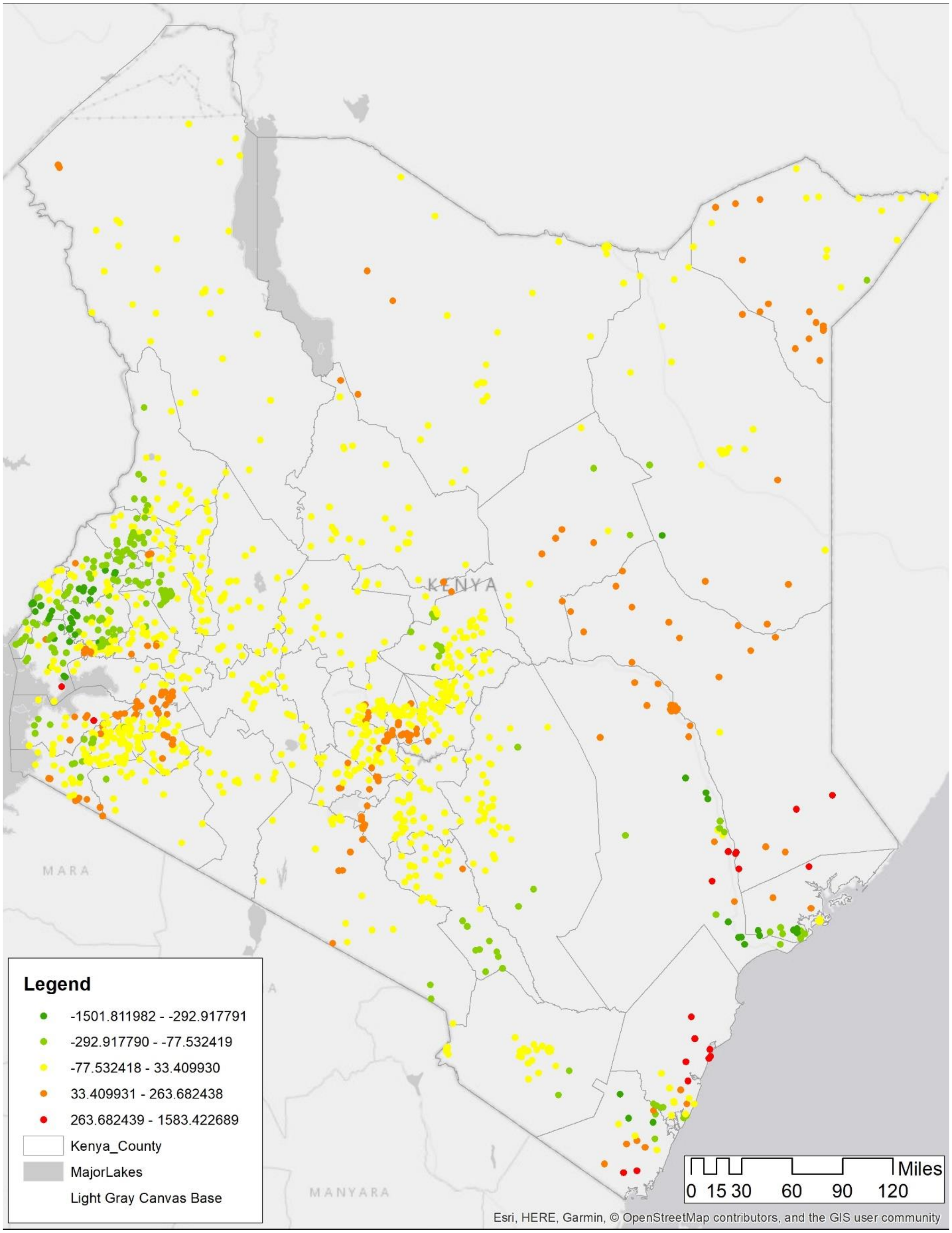

Figure S39. Map of GWR coefficients of elevation in 2005 (at DHS cluster)

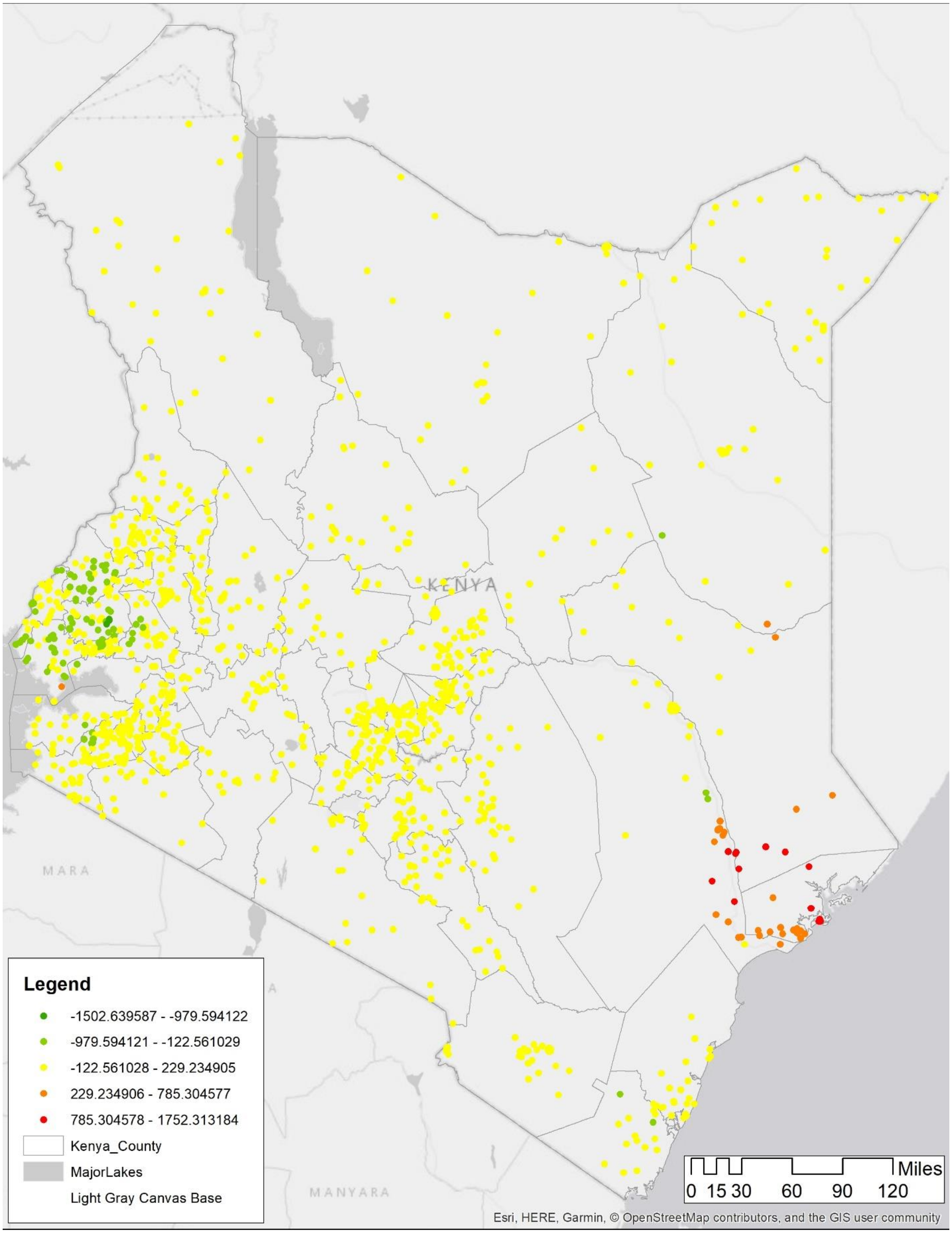

Figure S40. Map of GWR coefficients of elevation in 2010 (at DHS cluster)

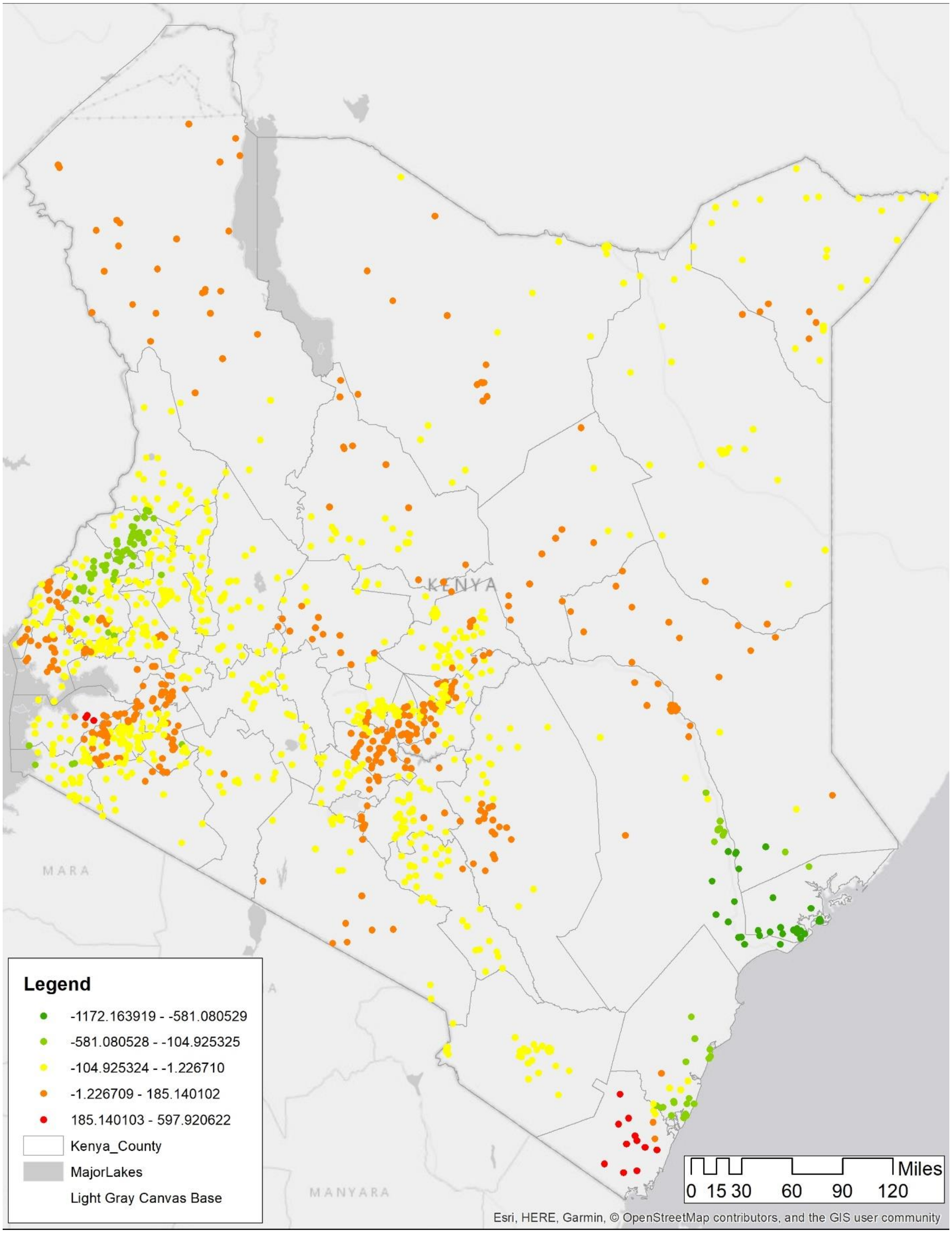

Figure S41. Map of GWR coefficients of elevation in 2015 (at DHS cluster)

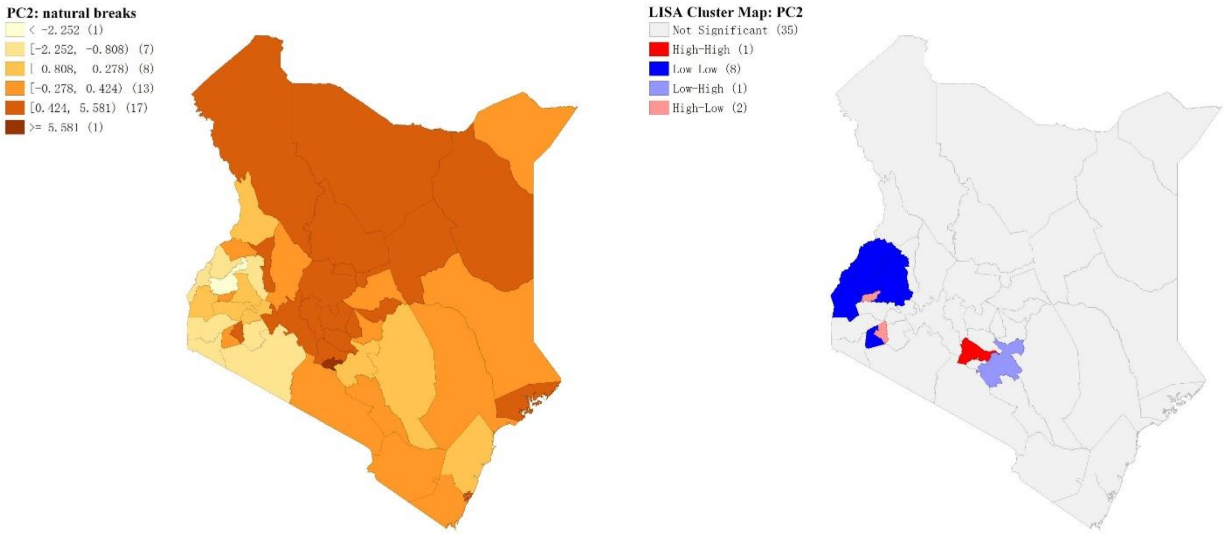

Figure S42. Left: Map of PC2 natural breaks; Right: Map of PC2 hotspots, cold spots and outliers (LISA)

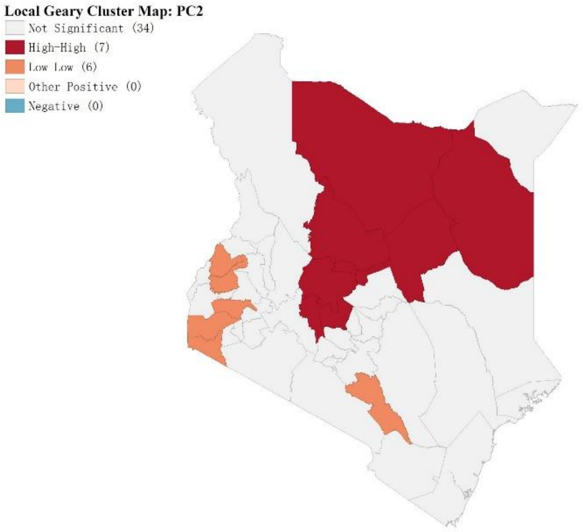

Figure S43. Map of Local Geary’s C Clusters, PC2
